# β-Amyloid discordance of cerebrospinal fluid and positron emission tomography imaging shows distinct spatial tau patterns

**DOI:** 10.1093/braincomms/fcac084

**Published:** 2022-03-31

**Authors:** Chenyang Jiang, Qingyong Wang, Siwei Xie, Zhicheng Chen, Liping Fu, Qiyu Peng, Ying Liang, Hongbo Guo, Tengfei Guo, for the Alzheimer’s Disease Neuroimaging Initiative

**Affiliations:** 1 Institute of Biomedical Engineering, Shenzhen Bay Laboratory, Shenzhen 518132, China; 2 Department of Neurology, University of Chinese Academy of Sciences-Shenzhen Hospital, Shenzhen 518107, China; 3 Institute of Chemical Biology, Shenzhen Bay Laboratory, Shenzhen 518132, China; 4 Department of Nuclear Medicine, China-Japan Friendship Hospital, 2 Yinghuayuan Dongjie, Beijing 100029, China; 5 Department of Nuclear Medicine, National Cancer Center/National Clinical Research Center for Cancer/Cancer Hospital & Shenzhen Hospital, Chinese Academy of Medical Sciences and Peking Union Medical College, Shenzhen 518116, China; 6 Department of Neurosurgery, Zhujiang Hospital, Southern Medical University, Guangzhou 510282, China; 7 Institute of Biomedical Engineering, Peking University Shenzhen Graduate School, Shenzhen 518055, China

**Keywords:** Alzheimer's disease, cerebrospinal fluid, positron emission tomography imaging, β-amyloid, Tau

## Abstract

Extracellular β-amyloid plaques and intracellular neurofibrillary tau tangles are the primary hallmarks of Alzheimer's disease. β-Amyloid pathology can be directly quantified by positron emission tomography imaging or indirectly by measuring the decrease of cerebrospinal fluid β-amyloid_42_/β-amyloid_40_ ratio. Although these two β-amyloid biomarkers may be considered interchangeable, they sometimes show discordance, particularly in early stage of Alzheimer's disease. Individuals with cerebrospinal fluid β-amyloid positive only or β-amyloid positron emission tomography positive only may be at early amyloidosis stage compared to those who are cerebrospinal fluid β-amyloid negative and β-amyloid positron emission tomography negative orcerebrospinal fluid β-amyloid positive and β-amyloid positron emission tomography positive. Besides, β-amyloid pathology may play an initiating role in Alzheimer's disease onset, leading to subsequent tau increases. However, it is still unclear whether individuals with different β-amyloid pathways have distinct spatial patterns of cortical tau tangles in early amyloidosis stage. In this study, we analyzed 238 cognitively unimpaired and 77 mild cognitive impairment individuals with concurrent (interval of acquisition <1 year) ^18^F-flortaucipir tau positron emission tomography, β-amyloid (^18^F-florbetapir or ^18^F-florbetaben) positron emission tomography and cerebrospinal fluid β-amyloid_42_ and β-amyloid_40_ and cerebrospinal fluid p-Tau_181_ and divided them into four different cerebrospinal fluid/positron emission tomography groups based on the abnormal status of cerebrospinal fluid β-amyloid_42_/β-amyloid_40_ (cerebrospinal fluid±) and β-amyloid positron emission tomography (±). We determined the cortical regions with significant tau elevations of different cerebrospinal fluid/positron emission tomography groups and investigated the region-wise and voxel-wise associations of tau positron emission tomography images with cerebrospinal fluid β-amyloid_42_/β-amyloid_40_, β-amyloid positron emission tomography and cerebrospinal fluid p-Tau/β-amyloid_40_ in early (cerebrospinal fluid positive/positron emission tomography negative and cerebrospinal fluid negative/positron emission tomography positive) and late (cerebrospinal fluid positive/positron emission tomography positive) amyloidosis stages. By compared to the cerebrospinal fluid negative/positron emission tomography negative individuals (Ref) without evidence of tau increase measured by cerebrospinal fluid or positron emission tomography, cerebrospinal fluid positive/positron emission tomography negative individuals showed higher tau in entorhinal but not in Braak_III/IV_ and Braak_V/VI_, whereas cerebrospinal fluid negative/positron emission tomography positive individuals had significant tau elevations in Braak_V/VI_ but not in entorhinal and Braak_III/IV_. In contrast, cerebrospinal fluid positive/positron emission tomography positive individuals showed significant tau increases in all the cortical regions than the Ref group. The voxel-wise analyses provided further evidence that lower cerebrospinal fluid β-amyloid_42_/β-amyloid_40_ was associated with higher tau in entorhinal, whilst higher β-amyloid positron emission tomography was related to higher tau in Braak_V/VI_ regions in early amyloidosis stage. Both lower cerebrospinal fluid β-amyloid_42_/β-amyloid_40_ and higher β-amyloid positron emission tomography were correlated with tau aggregation in all the Braak stages regions in late amyloidosis stage. These findings provide novel insights into the spatial patterns of cortical tau tangles in different amyloidosis stages of Alzheimer's disease, suggesting cerebrospinal fluid β-amyloid and β-amyloid positron emission tomography discordant groups may have distinct characteristics of cortical tau tangles in early amyloidosis stage.

## Teaser

Jiang *et al.* found that cerebrospinal fluid and positron emission tomography β-amyloid discordant individuals have distinct cortical tau deposition patterns in non-demented elderly adults, indicating different cerebrospinal fluid β-amyloid positive-first and positron emission tomography β-amyloid positive-first individuals may have initial tau tangles in distinct cortical regions in early amyloidosis stage of Alzheimer's disease.

## Graphical Abstract

**Graphical Abstract:**
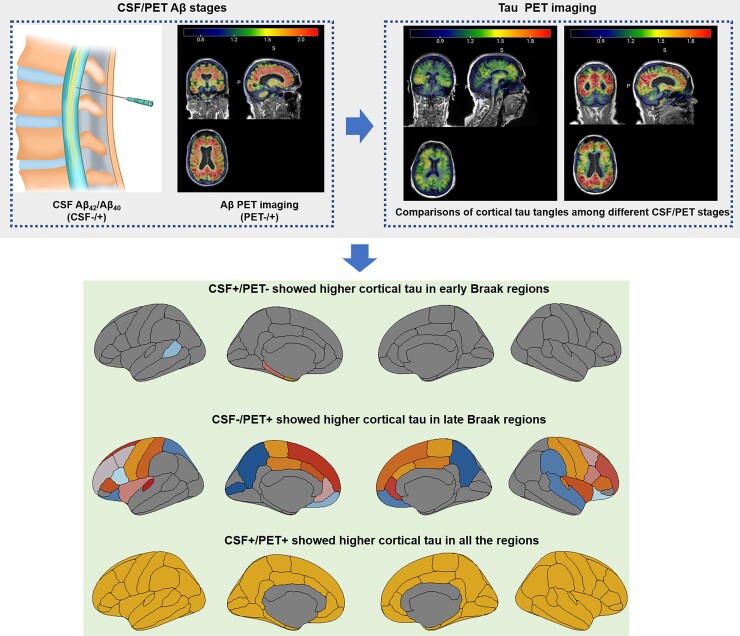

## Introduction

Extracellular β-amyloid (Aβ) plaques and intracellular neurofibrillary tau tangles are the primary hallmarks of Alzheimer's disease,^1^ and abnormal changes of Aβ pathology have been regarded as the earliest detectable change in Alzheimer's disease.^2,3^ Aβ pathology can be qualified *in vivo* by positron emission tomography (PET) imaging^4–6^ or indirectly by measuring the decrease of Aβ_42_/Aβ_40_ ratio in cerebrospinal fluid (CSF).^7^ The concordance and discordance of CSF Aβ and Aβ PET have been investigated cross-sectionally^8–21^ and longitudinally.^7,22^ Different groups^8,9,13–19,21^ have demonstrated that using CSF Aβ_42_/Aβ_40_ ratio showed better agreement with Aβ PET than using CSF Aβ_42_ alone in different cohorts. Nevertheless, either CSF Aβ (CSF Aβ_42_/Aβ_40_ ratio or CSF Aβ_42_ alone) or Aβ PET may become abnormal first.^7,13–15,17,22–31^ Together our laboratory^7^ and other group's findings,^11,22,32^ the individuals with CSF Aβ positive only (CSF+/PET−) or Aβ PET positive only (CSF−/PET+) may be at early amyloidosis stage compared to the individuals who are negative (CSF−/PET−) or positive (CSF+/PET+) at both CSF Aβ and Aβ PET. This may represent two different Aβ pathology progressing pathways (pathway1: CSF−/PET− → CSF+/PET− → CSF+/PET+; pathway2: CSF−/PET− → CSF−/PET+ → CSF+/PET+). Notably, the ‘Aβ pathway’ described in this study represents the different Aβ pathology progressing sequences rather than the biological mechanism of Aβ pathology.

Furthermore, Aβ pathology may play an initiating role in Alzheimer's disease onset, leading to subsequent tau increases^6,33,34^ or longitudinal tau changes.^3,35–37^ The Braak_I–VI_ stages^38^ have been proposed to characterize the spatial patterns of cortical neurofibrillary tau tangles based on the autopsy data. PET imaging studies^39–43^ provide further evidence that the cortical tau tangles may initially present in entorhinal cortex, following by spreading to the Braak_III/IV_ and Braak_V/VI_ cortical regions in the presence of substantial Aβ burden. However, it is still unclear whether individuals who are on the Alzheimer's continuum but with different Aβ pathways have distinct spatial patterns of cortical tau aggregation. Exploring the spatial distribution of cortical tau tangles is important for understanding the characteristics of Alzheimer's disease pathophysiology in different stages and may provide novel reference for designing anti-tau clinical trials of Alzheimer's disease.

In this study, we analyzed non-demented Alzheimer's Disease Neuroimaging Initiative (ADNI) participants who had concurrent (within 1 year) CSF Aβ_42_/Aβ_40_, phosphorylated tau (p-Tau), Aβ PET and tau PET data and divided them into four different CSF/PET amyloidosis stages based on the abnormal status of CSF Aβ_42_/Aβ_40_ and Aβ PET. In order to explore the spatial patterns of cortical tau tangles in different amyloidosis stages, we determined the cortical regions with significant tau elevation of different CSF/PET Aβ stages and investigated the region-wise and voxel-wise associations of tau PET images with CSF Aβ_42_/Aβ_40_, Aβ PET and CSF p-Tau/Aβ_40_ in early (CSF+/PET− and CSF−/PET+) and late (CSF+/PET+) amyloidosis stages.

## Materials and methods

### Participants

The data were obtained from the ADNI database (ida.loni.usc.edu). The ADNI study was approved by institutional review boards of all participating centres, and written informed consent was obtained from all participants or their authorized representatives. In this study, we identified 238 cognitively unimpaired (CU) and 77 mild cognitive impairment (MCI) ADNI participants with concurrent (interval of acquisition <1 year) ^18^F-flortaucipir (FTP) tau PET, amyloid [^18^F-florbetapir (FBP) or ^18^F-florbetaben (FBB)] PET, CSF Aβ_42_ and Aβ_40_ and CSF p-Tau. We divided these 315 participants into four CSF/PET groups according to Aβ positivity defined by the thresholds of CSF Aβ_42_/Aβ_40_ and Aβ PET as described below: CSF−/PET− (concordant Aβ negative), CSF+/PET− (discordant CSF Aβ positive), CSF−/PET+ (discordant Aβ PET positive) and CSF+/PET+ (concordant Aβ positive). In order to control for the influence of non-Alzheimer's related tauopathy, 43 CSF−/PET− participants with either abnormal CSF p-Tau/Aβ_40_^36^ or abnormal FTP tau PET (entorhinal or Temporal-metaROI^44^) were excluded from the CSF−/PET− group, and the rest of the CSF−/PET− group were defined as the reference (Ref) group.

### PET imaging and analysis

PET data were acquired in 5-min frames from 50 to 70 min (FBP), 90–110 min (FBB) and 75–105 min (FTP) post-injection, and more details are given elsewhere (http://adni-info.org). As described previously,^3^ FBP or FBB scans were coregistered to their corresponding baseline structural MRI scans. FreeSurfer (V5.3.0; https://surfer.nmr.mgh.harvard.edu/) was used to extract cortical Aβ tracer retention in 68 regions of interest (ROIs) defined by Desikan–Killiany atlas,^45^ and FBP or FBB standardized uptake value ratios (SUVRs) were calculated by referring regional florbetapir or florbetaben to that found in the whole cerebellum. A cortical summary COMPOSITE SUVR was created from a COMPOSITE cortical area, including frontal, cingulate, parietal and temporal regions.^46^

The FBB Aβ positivity of the COMPOSITE region was defined as SUVR  ≥  1.08, and FBP Aβ positivity of the COMPOSITE region was defined as SUVR  ≥  1.11. Finally, FBP and FBB SUVRs were converted to Centiloids using the equations Centiloid = (196.9 × SUVR_FBP_) – 196.03 and Centiloid = (159.08 × SUVR_FBB_) – 151.65.

FTP tau PET scans of 5 minutes × 4 frames were realigned, averaged and registered to the baseline MRI scan that was closest in time to the baseline FTP scan. FTP uptakes in 68 cortical ROIs defined by Desikan–Killiany atlas^45^ were extracted in native FTP tau PET space, and one composite Temporal-metaROI^44^ (including entorhinal, parahippocampal, fusiform, amygdala, inferior temporal and middle temporal) FTP SUVR was calculated by referring to a mean inferior cerebellar grey matter uptake.^47^ In order to evaluate tau deposition in different Braak neurofibrillary tau stages,^38^ we also calculated mean FTP SUVRs in Braak_III/IV_ and Braak_IV/VI_ that correspond to anatomical definitions of Braak stage III/IV (temporal/limbic) and V/VI (neocortical).^39^ The thresholds of entorhinal FTP SUVR and Temporal-meta ROI FTP SUVR were set as ≥1.21 and ≥1.25 respectively according to an ROC analysis using the Youden index classifying 280 Aβ– ADNI CU participants and 183 Aβ+ ADNI MCI and Alzheimer's disease patients as the endpoint as described previously^36^ and also in supplemental material (Supplemental Figs. 1–4).

For the voxel-wise analyses, FTP PET images were spatially normalized to the MNI space, intensity normalized at the voxel-wise level by a mean inferior cerebellar grey matter uptake^47^ and smoothed using a Gaussian kernal of 8 mm in SPM12 (Welcome Department of Imaging Neuroscience, London, UK).

### CSF biomarkers

CSF Aβ_40_, Aβ_42_ and p-Tau_181_ data were analyzed by the University of Pennsylvania ADNI Biomarker core laboratory using the fully automated Roche Elecsys and cobas e 601 immuno-assay analyzer system. CSF data (UPENN-BIOMK10_07_29_19.csv) were downloaded from the ADNI website. Considering that using a CSF p-Tau/Aβ_40_ ratio may reduce measurement error likely related to individual differences in CSF production rather than pathology, and improve associations with AD biomarkers compared to using CSF p-Tau alone,^36^ we decided to use CSF p-Tau/Aβ_40_ ratio to represent CSF tau in this study. The CSF Aβ_42_/Aβ_40_ ratio and CSF p-Tau/Aβ_40_ ratio^36^ were calculated, and their thresholds were defined as ≤0.054 and ≥0.0012 respectively according to the ROC analysis using the Youden index classifying 181 Aβ- CU participants and 163 Aβ+ cognitively impaired participants (MCI and Alzheimer's disease patients) as the endpoint as described in supplemental material (Supplemental Figs. 5–8).

### Statistical analysis

Normality of distributions was tested using the Shapiro–Wilk test and visual inspection of data histograms. Data are presented as median (interquartile range [IQR]) or number and percentage. Different CSF/PET groups were compared using a Mann–Whitney U test for continuous characteristics unless otherwise noted. We assessed categorical differences using Fisher's exact test. A false discovery rate (FDR) of 0.05 using Benjamini–Hochberg approach was employed for multi comparisons correction.

In order to investigate tau elevations of different CSF/PET groups, we used generalized linear model (GLM) to compare FTP SUVRs in 68 FreeSurfer-defined ROIs of CSF+/PET−, CSF−/PET+ and CSF+/PET+ groups with the Ref group (CSF−/PET− without evidence of elevated tau measured by either CSF or PET), controlling for age and sex. We also compared entorhinal FTP SUVR, Braak_III/IV_ FTP SUVR, Braak_V/VI_ FTP SUVR of different CSF/PET groups with the Ref group.

In addition, the voxel-wise FTP PET images of the CSF+/PET−, CSF−/PET+ and CSF+/PET+ groups were compared with the Ref group using two-sample *t*-test in SPM12, controlling for age and sex. The voxel-wise comparison between the CSF+/PET− group and the Ref group was presented using an uncorrected voxel threshold of *P* < 0.001, whilst the other comparisons were presented using an uncorrected voxel threshold of *P* < 0.001 and with family-wise error (FWE) corrected *P* < 0.05 at the cluster level.

As we described previously,^7^ both CSF+/PET− group and CSF−/PET+ group were defined as early amyloidosis stage whilst CSF+/PET+ individuals as late amyloidosis stage of Alzheimer's disease. In order to determine the associations of different Aβ biomarkers as well as CSF p-Tau/Aβ_40_ with cortical tau deposition of different Braak stages (entorhinal, Braak_III/IV_, and Braak_V/VI_) cortical regions in different amyloidosis stages, we used GLM model to investigate the association of FTP SUVRs in entorhinal, Braak_III/IV_ and Braak_V/VI_ with CSF Aβ_42_/Aβ_40_, Aβ PET and CSF p-Tau/Aβ_40_ in early (CSF+/PET− and CSF−/PET+ groups and including the CSF−/PET− group as the reference) and late (CSF+/PET+ group and including the CSF−/PET− group as the reference) amyloidosis stages separately, controlling for age and sex. We also investigated the voxel-wise association of FTP PET images with CSF Aβ_42_/Aβ_40_, Aβ PET and CSF p-Tau/Aβ_40_ in early and late amyloidosis stages separately, controlling for age and sex. The voxel-wise association between FTP SUVR images and CSF Aβ_42_/Aβ_40_ in early amyloidosis stage was presented using an uncorrected voxel threshold of *P* < 0.005, whilst the other comparisons were presented using an uncorrected voxel threshold of *P* < 0.001 and with FWE corrected *P* < 0.05 at the cluster level.

We noticed that one CSF+/PET− individual and one CSF+/PET+ individual had extremely high entorhinal FTP SUVRs; thus, we repeated all the analyses after removing them from the dataset. In addition, in order to control for the influence of those individuals around the thresholds of CSF Aβ_42_/Aβ_40_ and Aβ PET SUVR, we also repeated all the analyses after excluding the borderline participants who were within ±5% of the CSF Aβ_42_/Aβ_40_ and Aβ PET (SUVR) thresholds.

Statistical analyses were performed in the statistical programme R (v3.6.2, The R Foundation for Statistical Computing) unless otherwise noted.

### Data availability

All data used in the current study were obtained from the ADNI database (available at https://adni.loni.usc.edu).

## Results

### Demographics

The characteristics of the participants analyzed in this study can be found in Table 1. The majority were CSF−/PET− (Ref group) individuals (49.3%), with the second largest group CSF+/PET+ individuals (35.6%). The discordant groups CSF+/PET− individuals (6.6%) and CSF−/PET+ individuals (8.5%) had similar proportion. The CSF+/PET+ individuals had significantly older age, higher percentages of APOE ε4 carriers and MCI participants, lower CSF Aβ_42_/Aβ_40_, higher Aβ PET Centiloids and larger CSF p-Tau/Aβ_40_ than all the other groups. The CSF−/PET+ group had significantly older age than the Ref group as well. Both CSF+/PET− and CSF−/PET+ individuals had significantly lower CSF Aβ_42_/Aβ_40_ ratio, higher Aβ PET Centiloids and larger CSF p-Tau/Aβ_40_ ratio than the Ref group.

**Table 1:** Demographic characteristics of participants in different CSF/PET groups

CSF/PET groups	Ref	Early amyloidosis		Late amyloidosis
	CSF−/PET−	CSF+/PET−	CSF−/PET+	CSF+/PET+
Participants, *n* (%)	134 (49.3)	18 (6.6)	23 (8.5)	97 (35.6)
MCI, *n* (%)	26 (19.4)	6 (33.3)	2 (8.7)	**43** **(** **44.3)** ^a^
Age (years)	69.6 (8.8)	70.7 (14.9)	**72.5** (**7.3)**^b^	**75.1** (**10.5)**^c^
Sex, female. *n* (%)	86 (64.2)	8 (44.4)	13 (56.5)	56 (57.7)
APOE-ε4, *n* (%)	31 (23.1)	8 (44.4)	7 (30.4)	**64** (**66.0)**^d^
Education (years)	17.5 (2.0)	18.0 (2.0)	16.0 (3.0)	16.0 (4.0)
CSF Aβ_42_/Aβ_40_ ratio	0.09 (0.01)	**0.04** (**0.01)**^e,h^	**0.07** (**0.03)**^f^	**0.03** (**0.01)**^g,i,j^
Aβ PET Centiloids	4.44 (10.28)	**11.0** (**12.49)**^k^	**28.4** (**13.6)**^l,n^	**74.9** (**49.5)**^m,o,p^
CSF p-Tau/Aβ_40_ ratio	0.0009 (0.0002)	**0.0012** (**0.0003)**^q^	**0.0010 (0.0002)** ^r,t^	**0.0017 (0.0008)** ^s,u,v^

^a^ **Percentage of MCI**: CSF+/PET+ > CSF−/PET−: estimate = 3.29 [95% CI: 1.77, 6.22], *P* < 0.001; CSF+/PET+ > CSF−/PET+: estimate = 8.25 [95% CI: 1.85, 76.4], *P* < 0.01, Mann–Whitney U test.

**Age:** ^b^CSF−/PET+ > CSF−/PET−: estimate = 3.41 [95% CI: 0.63, 5.97], *P* = 0.048.

^c^ CSF+/PET+ > CSF−/PET−: estimate = 4.61 [95% CI: 2.62, 6.65], *P* < 0.001, Mann–Whitney U test.

^d^ **Percentage of APOE-ε4 Carriers**: CSF+/PET+ > CSF−/PET−: odds ratio = 6.38 [95% CI 3.47, 12.0], *P* < 0.001; CSF+/PET+ > CSF−/PET+: odds ratio = 4.37 [95% CI 1.52, 13.9], *P* = 0.012. Fisher exact test.

**CSF Aβ_42_/Aβ_40_ ratio**: ^e^CSF+/PET− < CSF−/PET−: estimate = −0.042 [−0.048, −0.037], *P* < 0.001.

^f^ CSF−/PET+ < CSF−/PET−: estimate = −0.02 [−0.020, −0.002], *P* < 0.014.

^g^ CSF+/PET+ < CSF−/PET−: estimate = −0.049 [−0.053, −0.046], *P* < 0.001.

^h^ CSF+/PET− < CSF−/PET+: estimate = −0.026 [−0.041, −0.018], *P* < 0.001.

^i^ CSF+/PET+ < CSF+/PET−: estimate = −0.007 [−0.012, −0.003], *P* = 0.001.

^j^ CSF+/PET+ < CSF−/PET+: estimate = −0.035 [−0.045, −0.029], *P* < 0.001; Mann–Whitney U test.

**Aβ PET Centiloids**: ^k^CSF+/PET− > CSF−/PET−: estimate = 6.93[2.61, 11.4], *P* = 0.002.

^l^ CSF−/PET+ > CSF−/PET−: estimate = 26.2 [22.3, 30.8], *P* < 0.001.

^m^ CSF+/PET+ > CSF−/PET−: estimate = 70.8 [64.0, 77.5], *P* < 0.001.

^n^ CSF−/PET+ > CSF+/PET−: estimate = 19.0 [13.9, 25.7], *P* < 0.001.

^o^ CSF+/PET+ > CSF+/PET−: estimate = 64.7 [50.1, 70.0], *P* < 0.001.

^p^ CSF+/PET+ > CSF−/PET+: estimate = 44.0 [29.0, 55.3], *P* < 0.001; Mann–Whitney U test.

**CSF p-Tau/Aβ_40_ ratio**: ^q^CSF+/PET− > CSF−/PET−: estimate = 0.0003[0.0002, 0.0004], *P* < 0.001.

^r^ CSF−/PET+ > CSF−/PET−: estimate = 0.0001[0.0000, 0.00002], *P* < 0.012.

^s^ CSF+/PET+ > CSF−/PET−: estimate = 0.0007[0.0006, 0.0008], *P* < 0.001.

^t^ CSF+/PET− > CSF−/PET+: estimate = 0.0002[0.0000, 0.0003], *P* = 0.003.

^u^ CSF+/PET+ > CSF+/PET−: estimate = 0.0005[0.0002, 0.0006], *P* < 0.001.

^v^ CSF+/PET+ > CSF−/PET+: estimate = 0.0007[0.0005, 0.0008], *P* < 0.001.

### Cortical tau elevations of different CSF/PET groups

**Figure 1:**
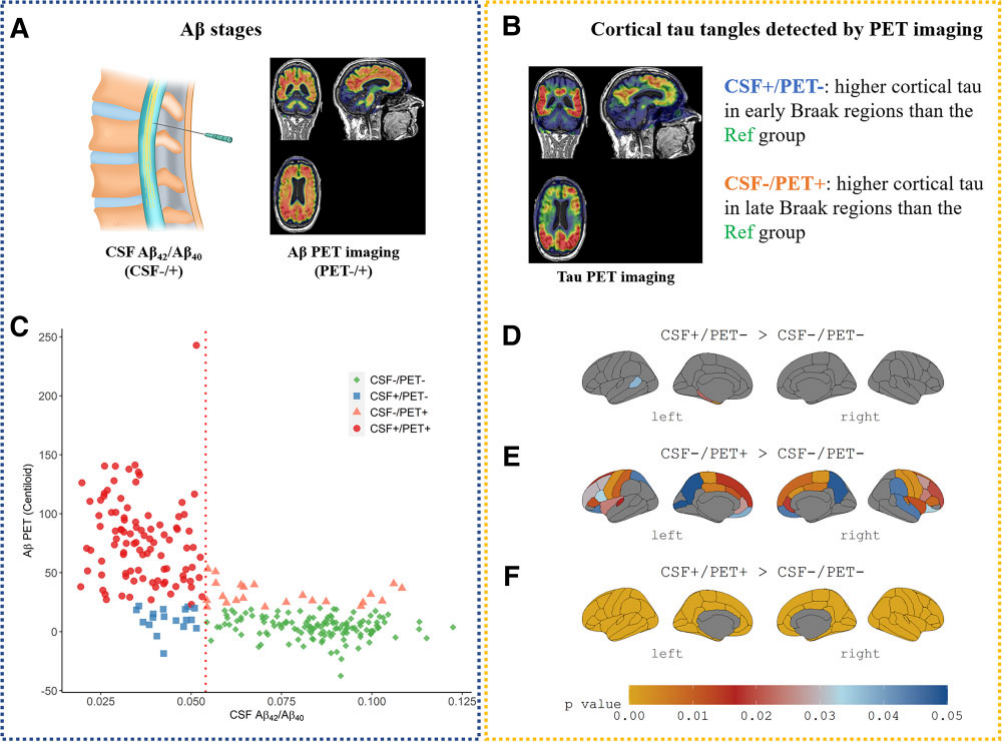
**Significant cortical tau elevation of different CSF/PET groups categorized by CSF Aβ and Aβ PET.** (**A**) Illustration of lumbar puncture and Aβ PET imaging. (**B**) Illustration of tau PET imaging. (**C**) CSF/PET groups defined by CSF Aβ_42_/Aβ_40_ and Aβ PET, and the vertical red dash line denotes the corresponding thresholds of CSF Aβ_42_/Aβ_40_ (0.054). (**D**, **E** and **F**) Significant cortical tau elevations of different CSF/PET groups compared with the CSF−/PET− group, and multiple comparisons correction was employed for 68 ROIs by using the Benjamini-Hochberg approach (FDR < 0.05) except for the comparison between CSF+/PET− group and Ref group.

As shown in Fig. 1, the CSF+/PET− group had significantly higher tau deposition in the left entorhinal and parahippocampal than the CSF−/PET− (Ref) group, and also in the left banks of superior temporal sulcus (BANKSSTS) although the effect size (*t* = 2.09, *P* = 0.037) was limited. In contrast, the CSF−/PET+ group showed significant tau increases in a few ROIs in Braak_IV_ stage (bilateral caudal anterior cingulate, rostral anterior cingulate, posterior cingulate, insula) and most of the ROIs in Braak_V/VI_ stage (except for frontal pole, pars orbitalis, lateral occipital, inferior parietal, BANKSSTS and cuneus) than the Ref group. The CSF+/PET+ group had significant tau elevation in all the cortical regions than the Ref group.

### Comparisons of tau deposition amongst different CSF/PET groups

**Figure 2:**
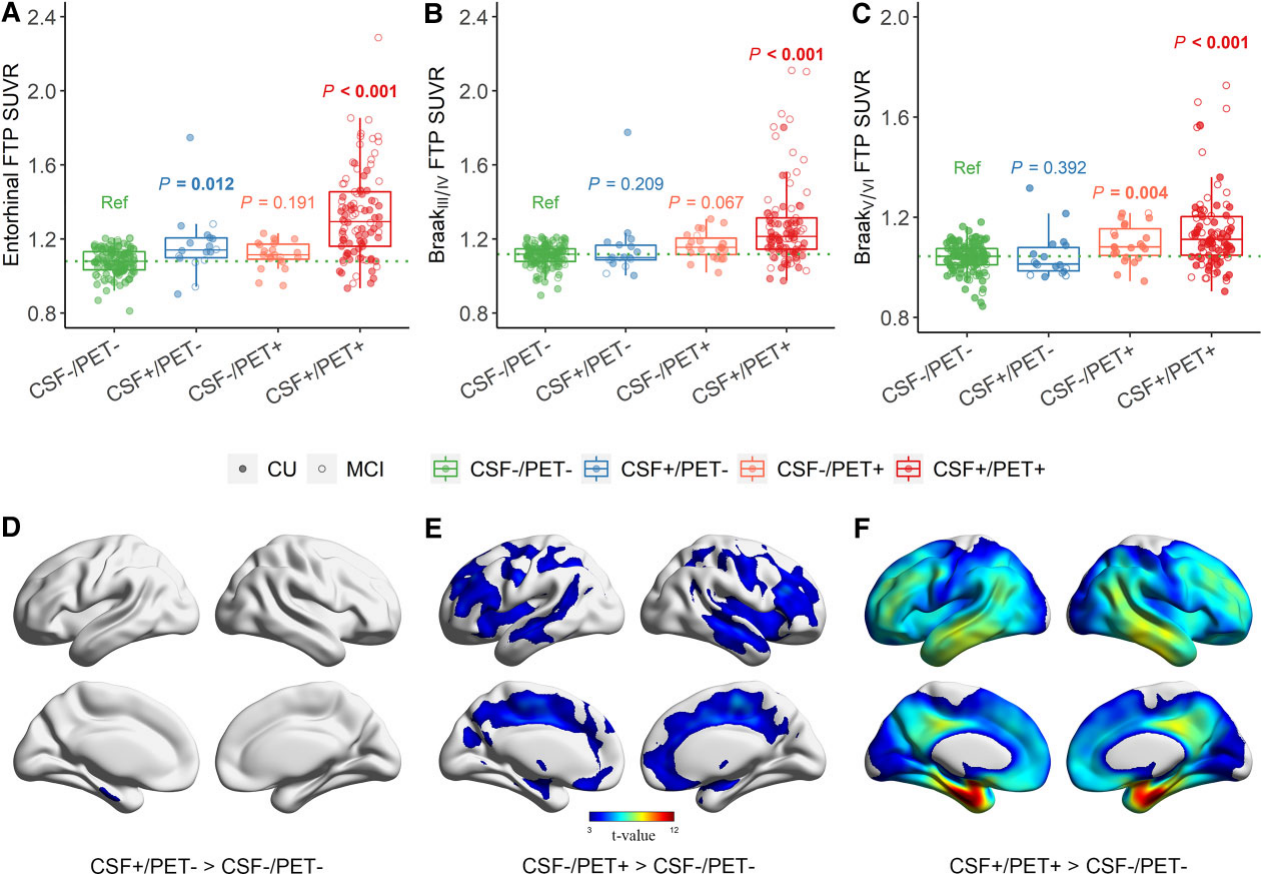
**Comparisons of cortical tau deposition among different CSF/PET groups.** Comparison of FTP SUVRs in (**A**) entorhinal, (**B**) Braak_III/IV_ and (**C**) Braak_V/VI_ among different CSF/PET groups. The boxplot whiskers extend to the lowest and highest data points within 1.5 times the IQR, from the lower and upper quartiles. The dots represent individual points of each CSF/PET group. Green dashed lines represent the median values of the reference (Ref) group. Values at the top of the bar indicate the *P* values of the comparisons with the Ref group. Voxel-wise comparisons of tau PET images of (**D**) CSF+/PET−, (**E**) CSF−/PET+ and (**F**) CSF+/PET+ with the Ref group. Two-sample *t*-tests, the comparison between CSF+/PET− group, and the Ref group was presented by using a threshold *P* < 0.001 at the voxel level, whilst the other comparisons were presented by using a threshold *P* < 0.001 at the voxel level and with FWE corrected *P* < 0.05 at the cluster level.

As illustrated in Fig. 2, the CSF+/PET− group had higher FTP SUVR in entorhinal (estimate = 0.10 [95% confidence interval (CI), 0.023 to 0.179], *P* = 0.012) but not in Braak_III/IV_ and Braak_V/VI_ than the Ref group. In contrast, the CSF−/PET+ group showed higher FTP SUVR in Braak_V/VI_ FTP SUVR (estimate = 0.069 [95% CI, 0.023 to 0.115], *P* = 0.004) but not in entorhinal and Braak_III/IV_ than the Ref group. The CSF+/PET+ group had higher FTP SUVR in entorhinal (estimate = 0.26 [95% CI, 0.219 to 0.305], *P* < 0.001), Braak_III/IV_ (estimate = 0.176 [95% CI, 0.136 to 0.215], *P* < 0.001) and Braak_V/VI_ FTP SUVR (estimate = 0.122 [95% CI, 0.094 to 0.149], *P* < 0.001) than the Ref group.

In the voxel-wise analysis, the CSF+/PET− group showed higher tau PET in the left entorhinal cortex and parahippocampal (Fig. 2D, *P* < 0.001 without cluster correction) than the Ref group, whereas the CSF−/PET+ group showed significant increases of FTP SUVRs in Braak_IV_ stage ROIs (bilateral caudal anterior cingulate, rostral anterior cingulate, insula, posterior cingulate and isthmus cingulate) and most of the Braak_V/VI_ stage ROIs (bilateral medial orbitofrontal, caudal middle frontal, rostral middle frontal, superior temporal, precuneus, postcentral, superior frontal, precentral and paracentral) (Fig. 2E, *P* < 0.001 with FWE *P* < 0.05 cluster correction). In addition, the CSF+/PET+ group showed significant (Fig. 2F, *P* < 0.001 with FWE cluster correction) elevated tau in most of the cortical regions except for pericalcarine and cuneus.

After removing two individuals with extremely high entorhinal FTP SUVRs, the CSF+/PET− group still had significantly higher tau deposition in the left entorhinal than the Ref group, and the results of other comparisons were substantially the same (Supplemental Fig. 9). Besides, the two discordant groups also showed distinct spatial cortical tau deposition, whilst we removed the individuals who were within ±5% of the CSF Aβ_42_/Aβ_40_ and Aβ PET thresholds (Supplemental Fig. 14), although more cortical regions (left entorhinal, parahippocampal, fusiform, inferior temporal, middle temporal, superior temporal and BANKSSTS) showed significant tau increases in CSF+/PET− group compared to the Ref group (Supplemental Fig. 15).

### Associations of cortical tau with CSF biomarkers and Aβ PET in early and late amyloidosis stages

**Figure 3:**
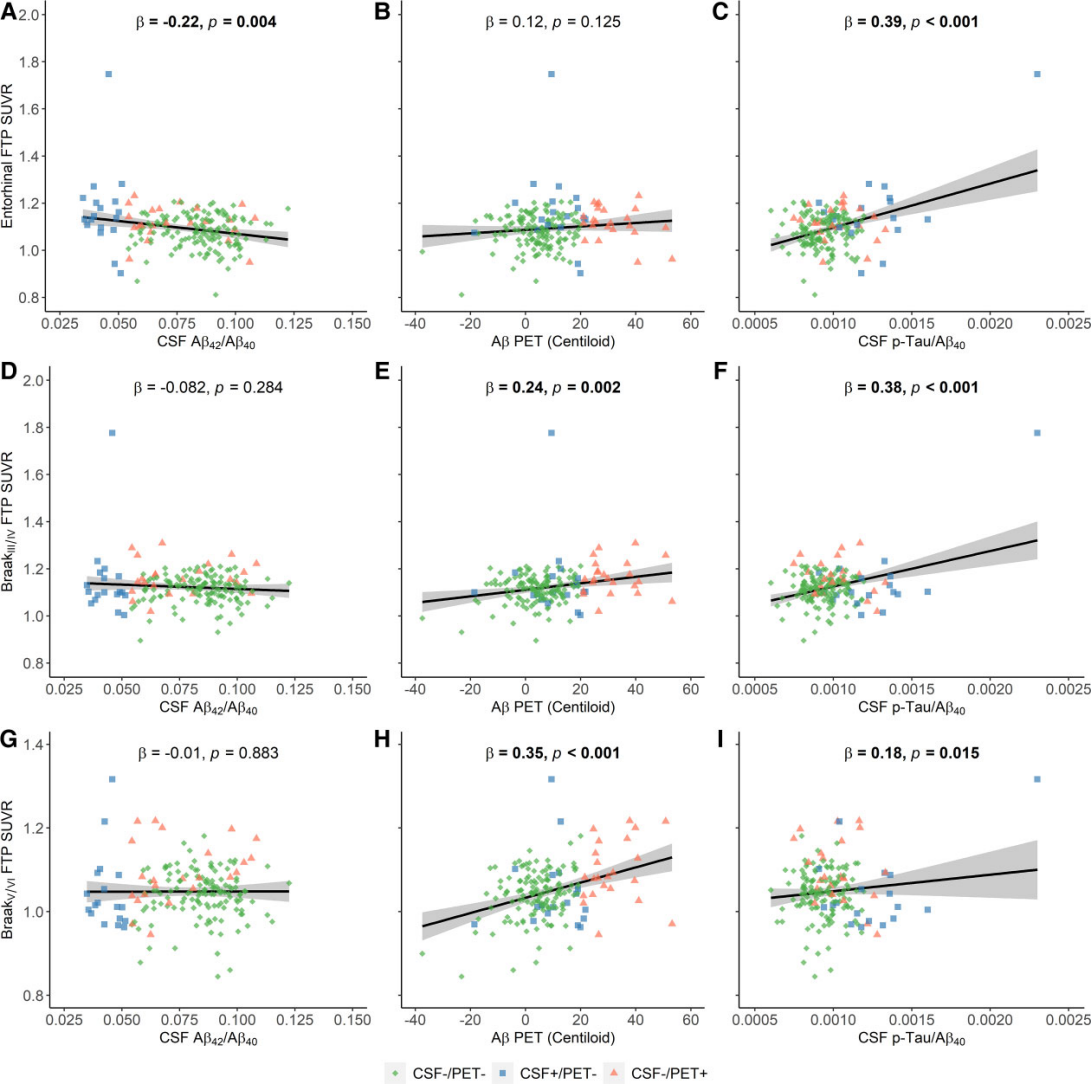
**The association of cortical tau deposition with CSF Aβ_42_/Aβ_40_, Aβ PET and CSF p-Tau/Aβ_40_ in early amyloidosis stage.** The association between entorhinal FTP SUVR and (**A**) CSF Aβ_42_/Aβ_40_, (**B**) Aβ-PET(Centiloid) and (**C**) CSF p-Tau/Aβ_40_. The association between Braak_III/IV_ FTP SUVR and (**D**) CSF Aβ_42_/Aβ_40_, (**E**) Aβ-PET(Centiloid) and (**F**) CSF p-Tau/Aβ_40_. The association between Braak_V/VI_ FTP SUVR and (**G**) CSF Aβ_42_/Aβ_40_, (**H**) Aβ-PET(Centiloid) and (**I**) CSF p-Tau/Aβ_40_.

In early amyloidosis stage, lower CSF Aβ_42_/Aβ_40_ was associated with higher FTP SUVR in entorhinal (standardized β (β_std_)    −0.22 [95% CI, −0.37 to −0.07], *P* = 0.004) but not in Braak_III/IV_ and Braak_V/VI_ (Fig. 3A, D and G), whereas higher Aβ PET was related to higher FTP SUVR in Braak_III/IV_ (Fig. 3E, β_std_ = 0.24 [95% CI, 0.09 to 0.38], *P* = 0.002) and Braak_V/VI_ (Fig. 3H, β_std_ = 0.35 [95% CI, 0.22 to 0.49], *P* < 0.001) but not in entorhinal cortex. However, we found positive association between CSF p-Tau/Aβ_40_ and tau PET in entorhinal, Braak_III/IV_ and Braak_V/VI_ (Fig. 3C, F and I).

**Figure 4:**
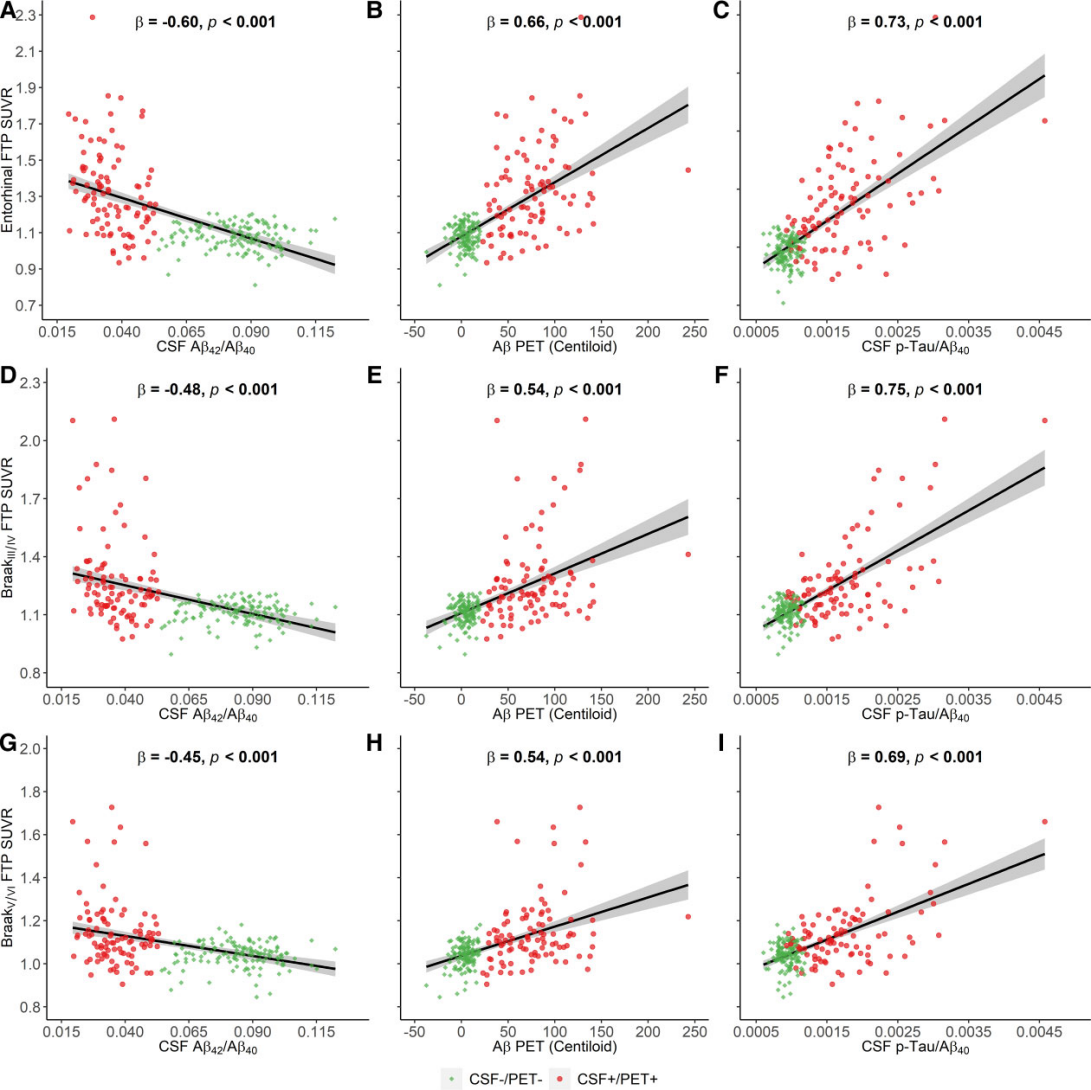
**The association of cortical tau deposition with CSF Aβ_42_/Aβ_40_, Aβ PET and CSF p-Tau/Aβ_40_ in late amyloidosis stage.** The association of entorhinal FTP SUVR with (**A**) CSF Aβ_42_/Aβ_40_, (**B**) Aβ-PET(Centiloid), and (**C**) CSF p-Tau/Aβ_40_. The association of Braak_III/IV_ FTP SUVR with (**D**) CSF Aβ_42_/Aβ_40_, (**E**) Aβ-PET(Centiloid), and (**F**) CSF p-Tau/Aβ_40_. The association of Braak_V/VI_ FTP SUVR with (**G**) CSF Aβ_42_/Aβ_40_, (**H**) Aβ-PET(Centiloid) and (**I**) CSF p-Tau/Aβ_40_.

In late amyloidosis stage, lower CSF Aβ_42_/Aβ_40_ was negatively related to higher FTP SUVR in entorhinal (Fig. 4A, β_std_ = −0.60 [95% CI, −0.71 to −0.49], *P* < 0.001), Braak_III/IV_ (Fig. 4D, β_std_ = −0.48 [95% CI, −0.59 to −0.36], *P* < 0.001) and Braak_V/VI_ (Fig. 4G, β_std_ = −0.45 [95% CI, −0.57 to −0.33], *P* < 0.001). Higher Aβ PET was positively related to higher FTP SUVR in entorhinal (Fig. 4B, β_std_ = 0.66 [95% CI, 0.55 to 0.76], *P* < 0.001), Braak_III/IV_ (Fig. 4E, β_std_ = 0.54 [95% CI, 0.43 to 0.66], *P* < 0.001) and Braak_V/VI_ FTP SUVR (Fig. 4H, β_std_ = 0.54 [95% CI, 0.42 to 0.65], *P* < 0.001). Higher CSF p-Tau/Aβ_40_ was positively associated with higher FTP SUVR in entorhinal (Fig. 4C, β_std_ = 0.73 [95% CI, 0.63 to 0.83], *P* < 0.001), Braak_III/IV_ (Fig. 4F, β_std_ = 0.75 [95% CI, 0.66 to 0.84], *P* < 0.001) and Braak_V/VI_ (Fig. 4I, β_std_ = 0.69 [95% CI, 0.59 to 0.79], *P* < 0.001).

After removing two individuals with extremely high entorhinal FTP SUVR, the significant association between tau PET and CSF p-Tau/Aβ_40_ in early amyloidosis stage disappeared, whilst the other associations retained (Supplemental Figs. 11 and 12). Besides, the results were substantially the same whilst we removed the borderline individuals (Supplemental Figs. 17 and 18).

### Voxel-wise analysis of cortical tau with CSF biomarkers and Aβ PET in early and late amyloidosis stages

**Figure 5:**
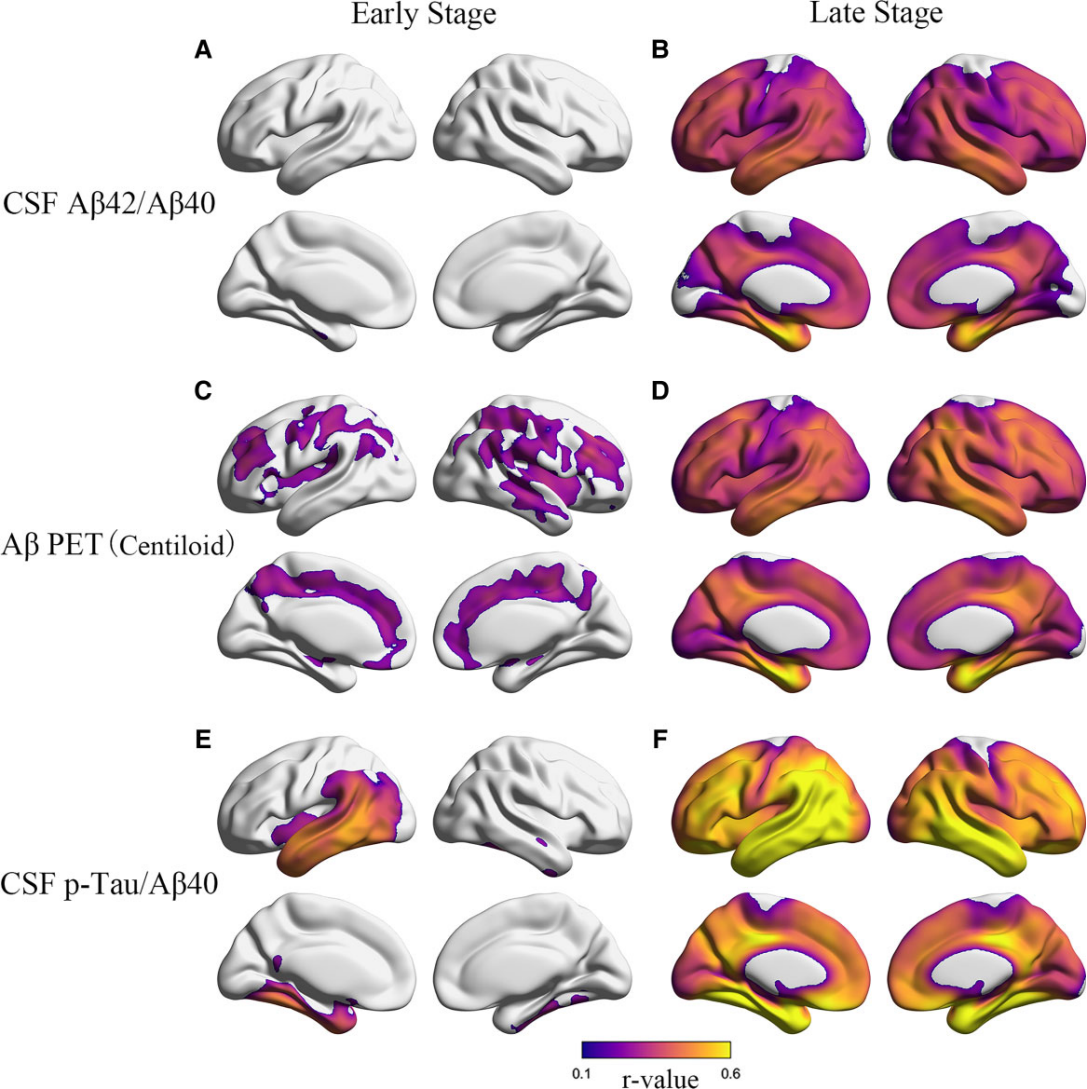
**Voxel-wise analyses of cortical tau with CSF biomarkers and Aβ PET in early and late amyloidosis stages.** Cortical regions with significant associations between FTP SUVR and (**A**) CSF Aβ_42_/Aβ_40_, (**C**) Aβ PET, (**E**) CSF p-Tau/Aβ_40_ and in early amyloidosis stage. The voxel-wise correlation between CSF Aβ_42_/Aβ_40_ and FTP tau PET was presented as *P* < 0.005 at the voxel level without cluster correction. The other voxel-wise correlation results were presented with using a threshold *P* < 0.001 at the voxel level and with FWE corrected *P* < 0.05 at the cluster level. Cortical regions with significant associations between FTP SUVR and (**B**) CSF Aβ_42_/Aβ_40_, (**D**) Aβ PET, (**F**) CSF p-Tau/Aβ_40_ and in the late amyloidosis stage. Results are shown using a threshold *P* < 0.001 at the voxel level and with FWE corrected *P* < 0.05 at the cluster level.

In early amyloidosis stage, lower CSF Aβ_42_/Aβ_40_ was significantly associated with higher FTP SUVR in the left entorhinal cortex (Fig. 5A), higher Aβ PET Centiloid was significantly related to higher FTP SUVRs in right insula and bilateral cingulate cortex, paracentral, frontal and parietal cortices (Fig. 5C), and CSF p-Tau/Aβ_40_ showed significant positive association with FTP SUVRs in left entorhinal, parahippocampal, fusiform, inferior temporal, middle temporal and BANKSSTS (Fig. 5E). In contrast, CSF Aβ_42_/Aβ_40_, Aβ PET and CSF p-Tau/Aβ_40_ all showed significant relation with FTP SUVRs in all the Braak ROIs, and the strongest association was found between FTP SUVR and CSF p-Tau/Aβ_40_ in late amyloidosis stage (Fig. 5B, D and F). Besides, the strongest associations with tau PET for all the biomarkers were found in early Braak ROIs (entorhinal and Braak_III/IV_) in late amyloidosis stage.

After removing two individuals with extremely high entorhinal FTP SUVRs, the significant association between tau PET and CSF p-Tau/Aβ_40_ in early amyloidosis stage disappeared, whilst the other associations retained (Supplemental Fig. 13). Besides, the results were substantially the same whilst we removed the borderline individuals (Supplemental Fig. 19).

## Discussion

In this study, we investigated the cortical tau deposition measured by tau PET imaging of different amyloidosis stages defined by CSF Aβ_42_/Aβ_40_ and Aβ PET in non-demented elderly adults. Compared to CSF Aβ_42_/Aβ_40_ negative and Aβ PET negative (CSF−/PET−) individuals without tau increase in CSF or cortex, we found individuals with abnormal CSF Aβ_42_/Aβ_40_ only (CSF+/PET−) showed higher tau in entorhinal but not in Braak_III/IV_ and Braak_V/VI_, whereas individuals with abnormal Aβ PET only (CSF−/PET+) had significant tau elevations in Braak_V/VI_ but not in entorhinal and Braak_III/IV_. The voxel-wise analyses provided further evidence that lower CSF Aβ_42_/Aβ_40_ was associated with higher tau in entorhinal, whilst higher Aβ PET was related to higher tau in Braak_V/VI_ ROIs in early amyloidosis stage (CSF+/PET− and CSF−/PET+). In contrast, individuals with abnormal CSF Aβ_42_/Aβ_40_ and abnormal Aβ PET (CSF+/PET+) had significant tau elevations in all the Brank ROIs, and both lower CSF Aβ_42_/Aβ_40_ and higher Aβ PET were correlated with higher tau in entorhinal, Braak_III/ IV_ and Braak_V/VI_ in late amyloidosis stage. These findings provide novel insights into understanding the cortical tau aggregation in different amyloidosis stages of Alzheimer's disease, suggesting CSF Aβ and Aβ PET discordant individuals may have initial tau tangles in distinct cortical regions in early amyloidosis stage of Alzheimer's disease.

In line with previous studies,^7,8,16^ we found CSF+/PET− individuals (CSF-first Aβ pathway) and CSF−/PET+ individuals (PET-first Aβ pathway) had similar proportion, and both of them had more Aβ pathology measured by either CSF or PET imaging than the CSF−/PET− individuals, supporting that CSF Aβ and Aβ PET may measure different features of Aβ pathology^7,24,48^ and two distinct Aβ pathways^22^ exist in early amyloidosis stage of Alzheimer's disease. Furthermore, both CSF+/PET− and CSF−/PET+ individuals had higher CSF p-Tau/Aβ_40_ ratios than the CSF−/PET− individuals, implying that the early abnormal tau increases in CSF^36,49,50^ are detectable in early amyloidosis stage. Notably, the CSF+/PET− individuals also showed higher CSF p-Tau/Aβ_40_ ratio than the CSF−/PET+ individuals. Together, these findings suggest that the CSF-first Aβ pathway (CSF−/PET− → CSF+/PET− → CSF+/PET+) may have more soluble Alzheimer's disease pathophysiology, which typically present in early stage of Alzheimer's disease,^7,36,49,50^ than the PET-first Aβ pathway (CSF−/PET− → CSF−/PET+ → CSF+/PET+).

Whilst CSF measurement of p-Tau provides complementary early tau increase,^36,49,50^ PET imaging offers spatial information on where tau deposits. Notably, our group^36^ and other laboratories^49,50^ very recently observed evidence that CSF p-Tau may detect early tau increase than the tau PET imaging, which was also supported by postmortem studies.^51–53^ Importantly, both dichotomous and continuous analyses showed that the CSF-first Aβ pathway had Aβ-related tau increase in entorhinal cortex, which has been regarded as the earliest cortical region of tau aggregation.^38–43^ In contrast, the PET-first Aβ pathway showed significant Aβ-related tau increase in Braak_V/VI_ but not in entorhinal and Braak_III/IV_. In concordance with our findings, one recent important ADNI study^32^ also found that the CSF+/PET− and CSF−/PET+ individuals have numerically (not significant) higher and lower entorhinal tau measured around 5 year post-baseline CSF Aβ and Aβ PET than the CSF−/PET− individuals respectively, although they used CSF Aβ_42_ to define CSF Aβ status, which may be less reliable than CSF Aβ_42_/Aβ_40_ used in this study. They also found CSF−/PET+ but not CSF+/PET− individuals had significant tau increase in Braak_V/VI_ but not in entorhinal and Braak_III/IV_, which was consistent with our findings. Together with our findings and previous study,^32^ it is probably that individuals with PET-first Aβ pathway may not have tau increases in entorhinal and Braak_III/IV_ cortical regions due to their lower CSF p-Tau, which plays an important role in tau spreading in cortical regions of early Braak stages (entorhinal and Braak_III/IV_).^36,49,50^ The voxel-wise results provide further evidence to support the notion that CSF Aβ_42_/Aβ_40_ may be related to tau aggregation in entorhinal cortex whereas cortical Aβ burden correlates with elevated tau in cortical regions of Braak_V/VI_ stage in early amyloidosis stages. Notably, the CSF−/PET+ individuals had smaller CSF p-Tau/Aβ_40_ ratio than the CSF+/PET− individuals, suggesting we may not be able to use CSF p-Tau biomarker to represent complementary Braak_V/VI_ tau increase in CSF−/PET+ individuals.

Our group^7^ and other laboratory^11,54^ previously observed that CSF+/PET− individuals were accumulating cortical Aβ burden with a similar rate to the CSF+/PET+ individuals, and will become CSF+/PET+ in future. Besides, the CSF-first Aβ pathway has been observed more frequent than the PET-first Aβ pathway according to the previous reports.^9,13–15,17^ In this study, we found similar proportion of CSF and PET Aβ discordant groups, but our previous longitudinal analyses^7^ also support that CSF Aβ may become abnormal earlier than Aβ PET. Consequently, it is probably that the CSF-first Aβ pathway may represent the typical evolution of Alzheimer's disease which shows Alzheimer's typical tau spreading pattern,^38–43^ whilst the PET-first Aβ pathway may have cortical Aβ-burden related hippocampal-sparing elevated tau in early amyloidosis stage of Alzheimer's disease. The Temporal-metaROI (entorhinal, amygdala, parahippocampal, fusiform, inferior temporal and middle temporal)^44^ composite regions have been commonly used to detect Alzheimer's-related tau deposition in human brain.^3,55–60^ However, our findings suggested that we may not be able to use Temporal-metaROI regions to capture the cortical tau increase in early amyloidosis stage among these individuals who have a PET-first Aβ pathway, implying different cortical regions should be selected to detect early tau increase in early Alzheimer's disease.

In contrast, we found CSF Aβ positive and Aβ PET positive (CSF+/PET+, late amyloidosis stage) individuals showed significant tau increases in all the cortical regions than the control group, and the highest tau elevations were found in the Temporal-metaROI^44^ composite regions. Furthermore, the voxel-wise analyses revealed that lower CSF Aβ_42_/Aβ_40_, higher Aβ PET, and larger CSF p-Tau/Aβ_40_ ratio were related to significant tau elevations in all the cortical regions and with Temporal-metaROI^44^ composite regions showing the strongest association. These findings suggest that it is reasonable to use Temporal-metaROI regions to detect cortical tau increase in CSF and PET Aβ concordant individuals.

## Limitations

This study has several limitations. First, as CSF Aβ and Aβ PET have a very high agreement, the sample sizes of the discordant CSF/PET Aβ groups with concurrent tau PET imaging were relatively small and the results need to be replicated in a larger cohort. To the best of our knowledge, there is currently no larger cohort available with the measurements needed for this analysis. Second, the ADNI participants overall are a highly selected sample, recruited to reflect the exclusionary criteria and types of individuals likely to participate in clinical trials; thus, it would be extremely useful to validate these findings in other aging cohorts. Third, our analyses were limited to cross-sectional PET measured with FTP, which may need to be replicated using longitudinal data and other PET ligands. Forth, the threshold of CSF Aβ_42_/Aβ_40_ was defined on the basis of the ADNI database, which requires validation with other databases.

## Conclusion

In conclusions, we found that CSF and PET Aβ discordant individuals have distinct cortical tau deposition patterns in non-demented elderly adults. Recent studies^61–64^ suggest that Alzheimer's disease may have distinct biological features of tau spreading patterns, which are important for explaining the heterogeneity of tau-related neurodegeneration and cognitive decline and the design of anti-tau clinical trials. Our findings are useful for understanding the subtypes of tau spreading patterns in Alzheimer's disease and provide novel reference for cortical tau detection in individuals who are at early amyloidosis stage.

## Supplementary Material

fcac084_Supplementary_Data

## Abbreviations

Aβ =: β-amyloid
AD =: Alzheimer's disease
ADNI =: Alzheimer's Disease Neuroimaging Initiative
BANKSSTS =: the banks of superior temporal sulcus
CI =: confidence interval
CSF =: cerebrospinal fluid
CU =: cognitively unimpaired
FBB =: ^18^F-florbetaben
FBP =: ^18^F-florbetapir
FDR =: false discovery rate
FTP =: ^18^F-flortaucipir
FWE =: family wise error
GLM =: Generalized Linear Model
IQR =: interquartile range
MCI =: mild cognitive impairment
PET =: positron emission tomography
p-Tau =: phosphorylated tau
Ref =: reference
ROI =: region of interest
SUVR =: standardized uptake value ratio
