# Supplementary material for: β-Amyloid discordance of cerebrospinal fluid and positron emission tomography imaging shows distinct spatial tau patterns
Source: Brain Commun. 2022 Mar 31;4(2):fcac084. doi: 10.1093/braincomms/fcac084 (PMC9014538; doi:10.1093/braincomms/fcac084)
Supplement: fcac084_Supplementary_Data [file fcac084_supplementary_data.pdf]

Supplementary Material - The discordance of β-amyloid measured by CSF and PET imaging shows distinct spatial cortical tau tangles

Cutoffs of tau PET 2
Cutoff of temporal meta ROI FTP SUVR...2
Cutoff of Entorhinal FTP SUVR...4
Cutoffs of CSF $A\beta_{42}/A\beta_{40}$ ratio and CSF p-Tau/$A\beta_{40}$ ratio 6
Analyses after excluding two individuals with high entorhinal tau..............................10
Sensitivity analysis...15

Cutoffs of tau PET

Cutoff of temporal meta ROI FTP SUVR

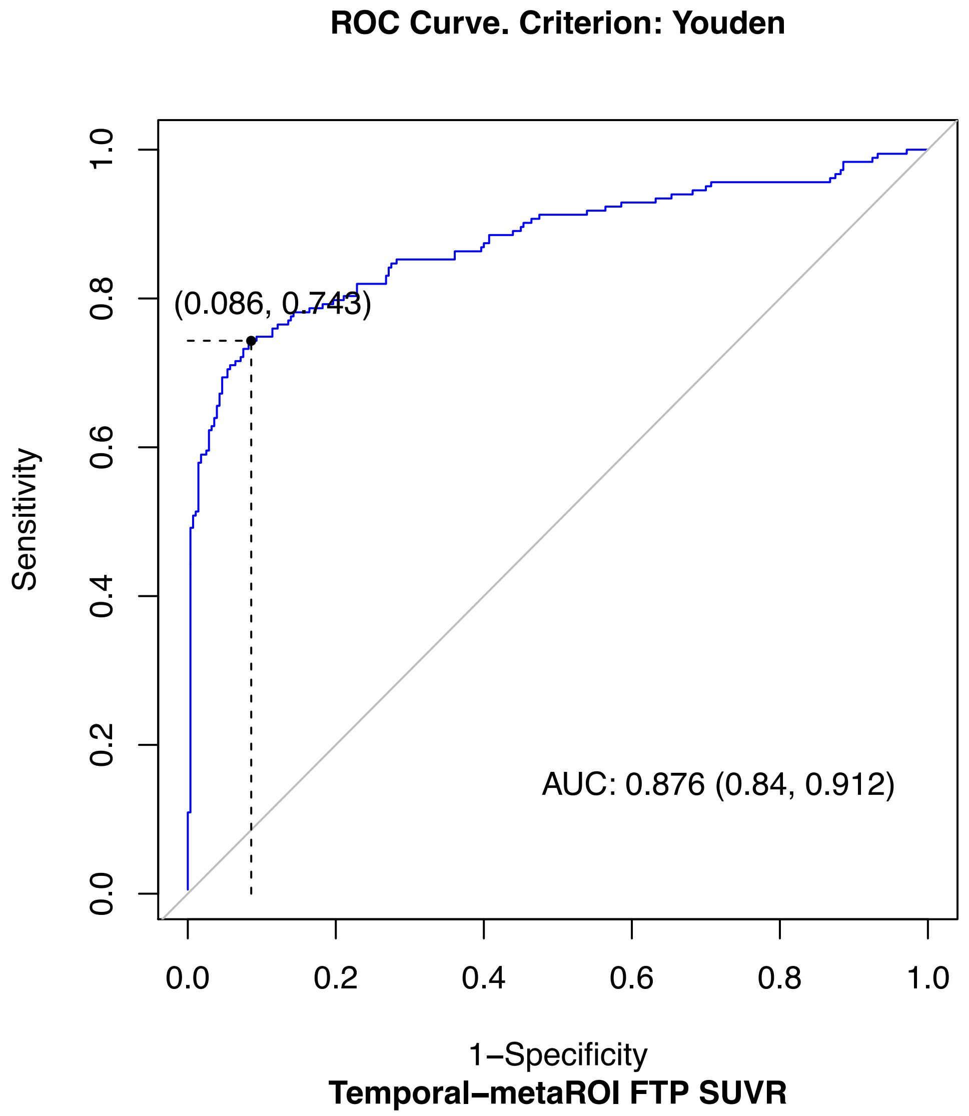

Supplementary figure 1. *The ROC analysis using the Youden index classifying 280 Aβ- ADNI cognitively unimpaired (CU) participants and 183 Aβ+ ADNI MCI and AD patients as the endpoint to define the cutoff ⩾1.25 for Temporal-metaROI FTP SUVR. AUC: 0.876 (95%CI, 0.84, 0.912).*

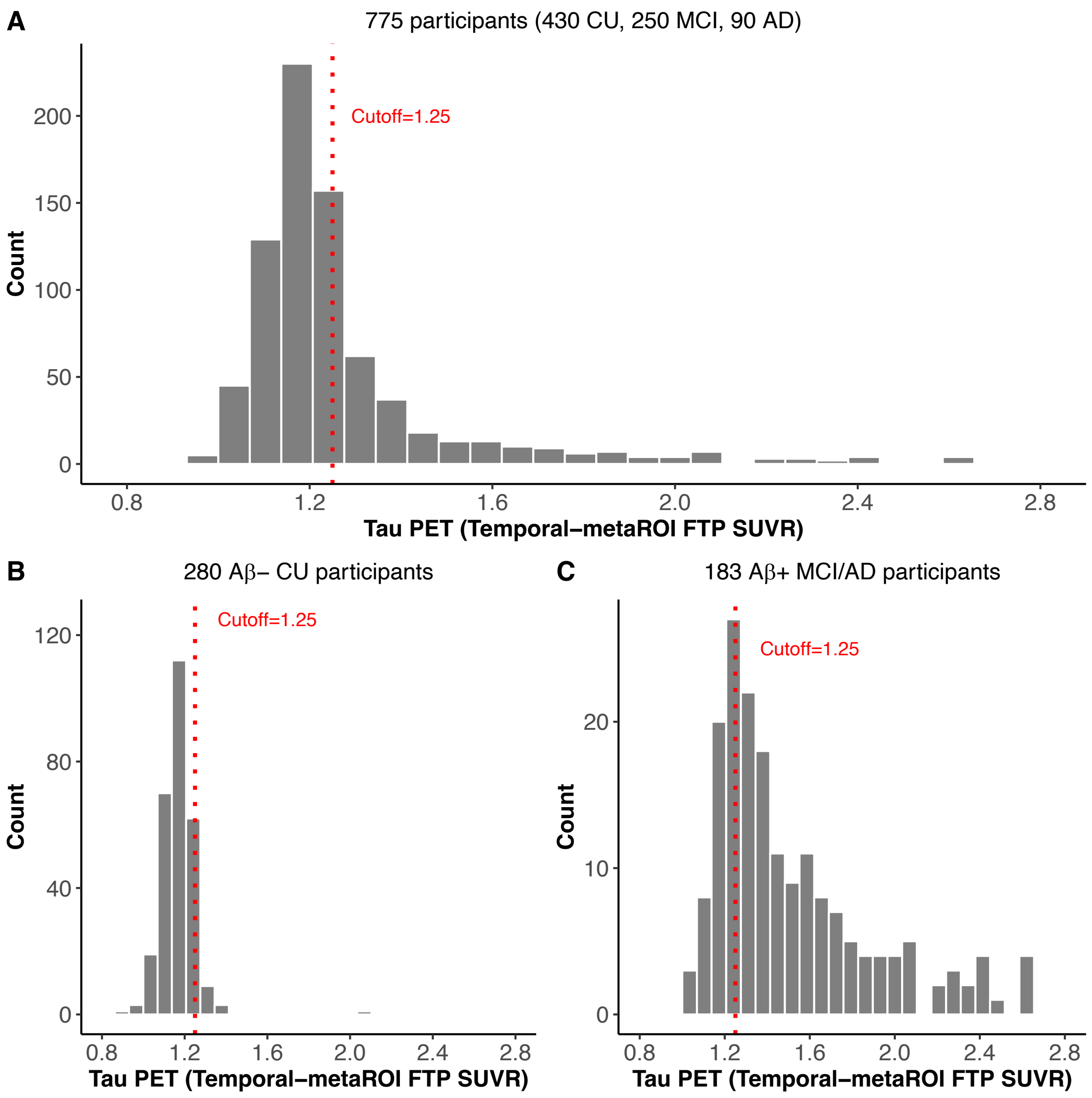

Supplementary figure 2. *Histograms of Temporal-metaROI FTP SUVRs of (A) all 775 ADNI participants, (B) 280 Aβ- ADNI CU participants and (C) 183 Aβ+ ADNI MCI and AD patients with tau PET scan. Red dotted line is the cutoff of Temporal-metaROI FTP SUVR 1.25.*

Cutoff of Entorhinal FTP SUVR

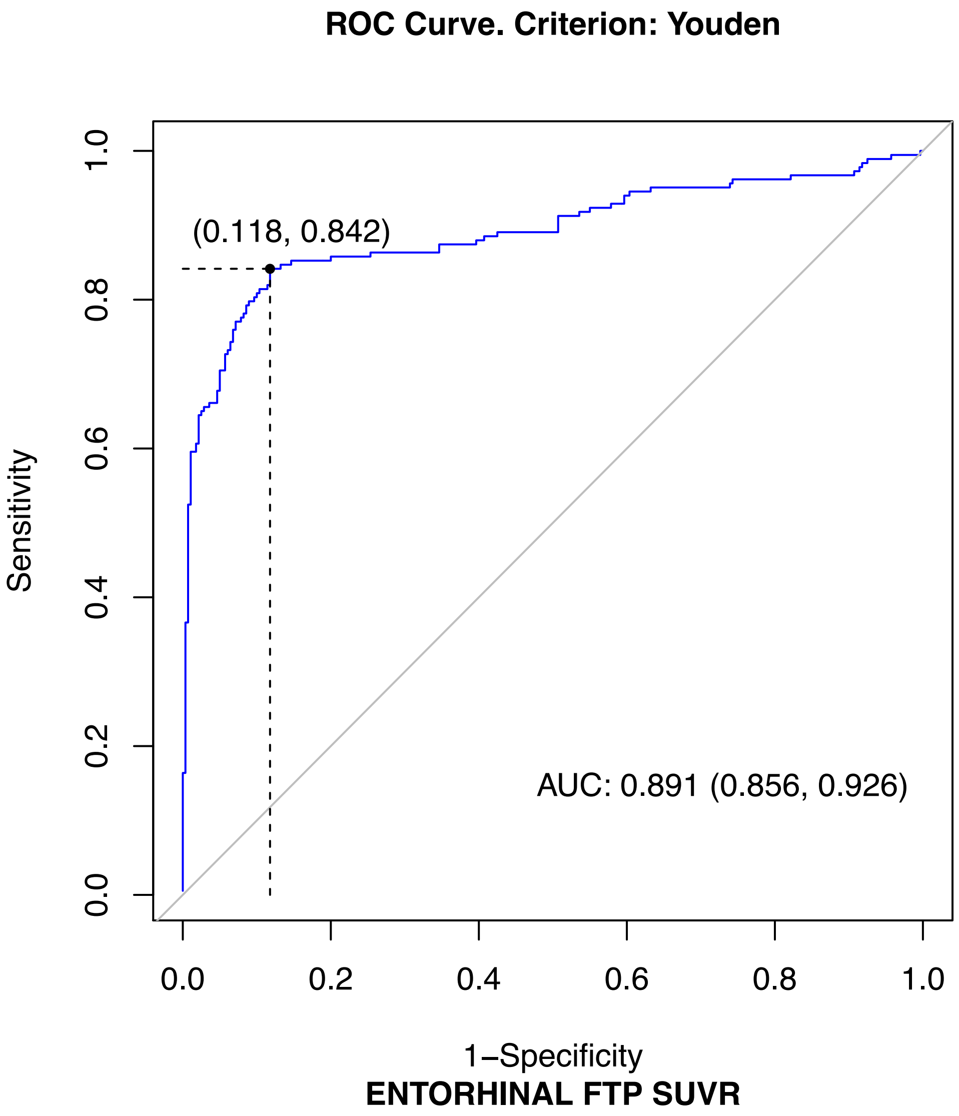

Supplementary figure 3. *The ROC analysis using the Youden index classifying 280 Aβ- ADNI CU participants and 183 Aβ+ ADNI MCI and AD patients as the endpoint to define the cutoff ⩾1.21 for entorhinal FTP SUVR. AUC: 0.891 (95%CI, 0.856, 0.926).*

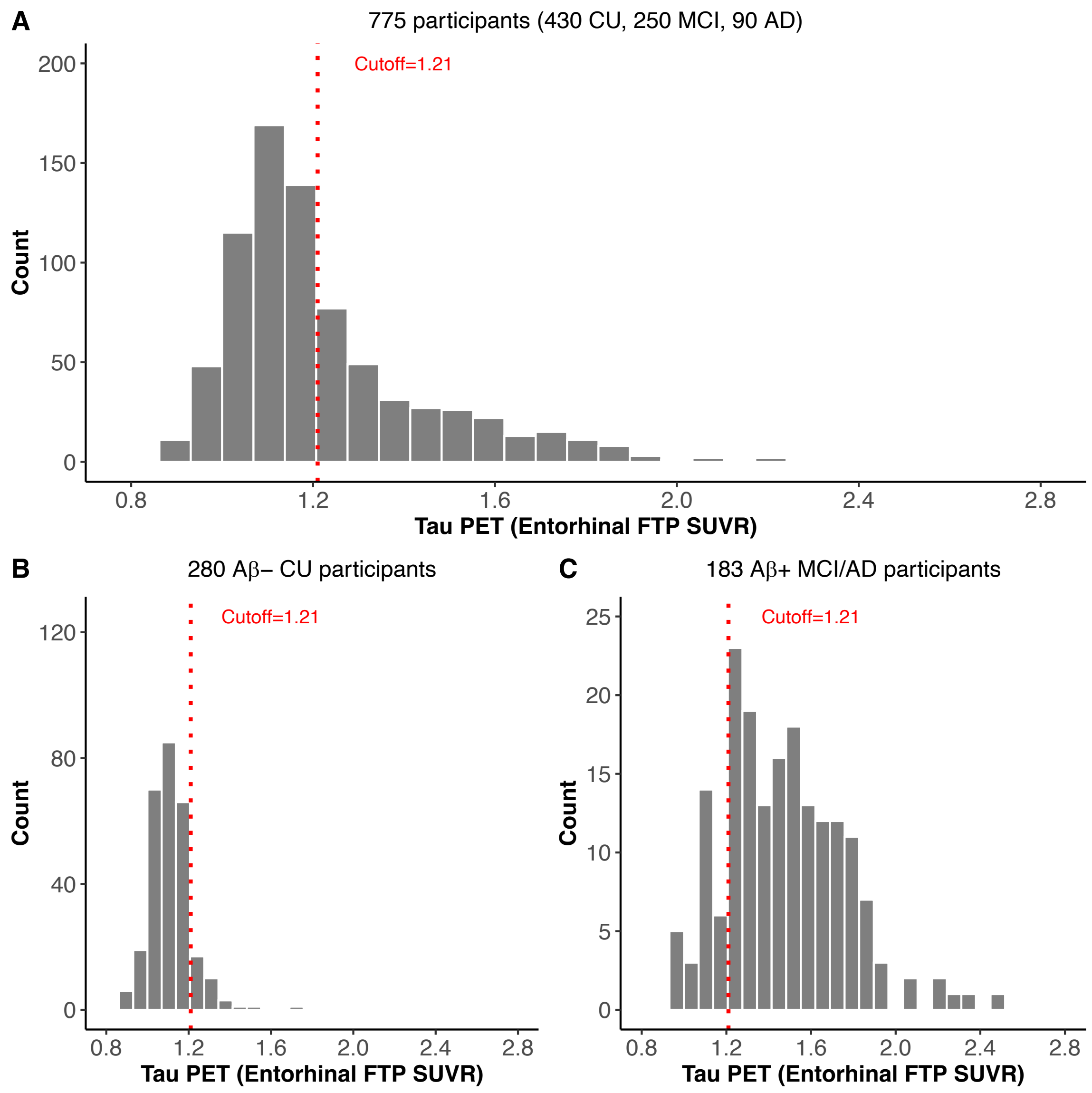

Supplementary figure 4. *Histograms of entorhinal FTP SUVRs of (A) all 775 ADNI participants, (B) 280 Aβ- ADNI CU participants and (C) 183 Aβ+ ADNI MCI and AD patients with tau PET scan. Red dotted line is the cutoff of entorhinal FTP SUVR 1.21.*

Cutoffs of CSF Aβ_{42}/Aβ_{40} ratio and CSF p-Tau/Aβ_{40} ratio

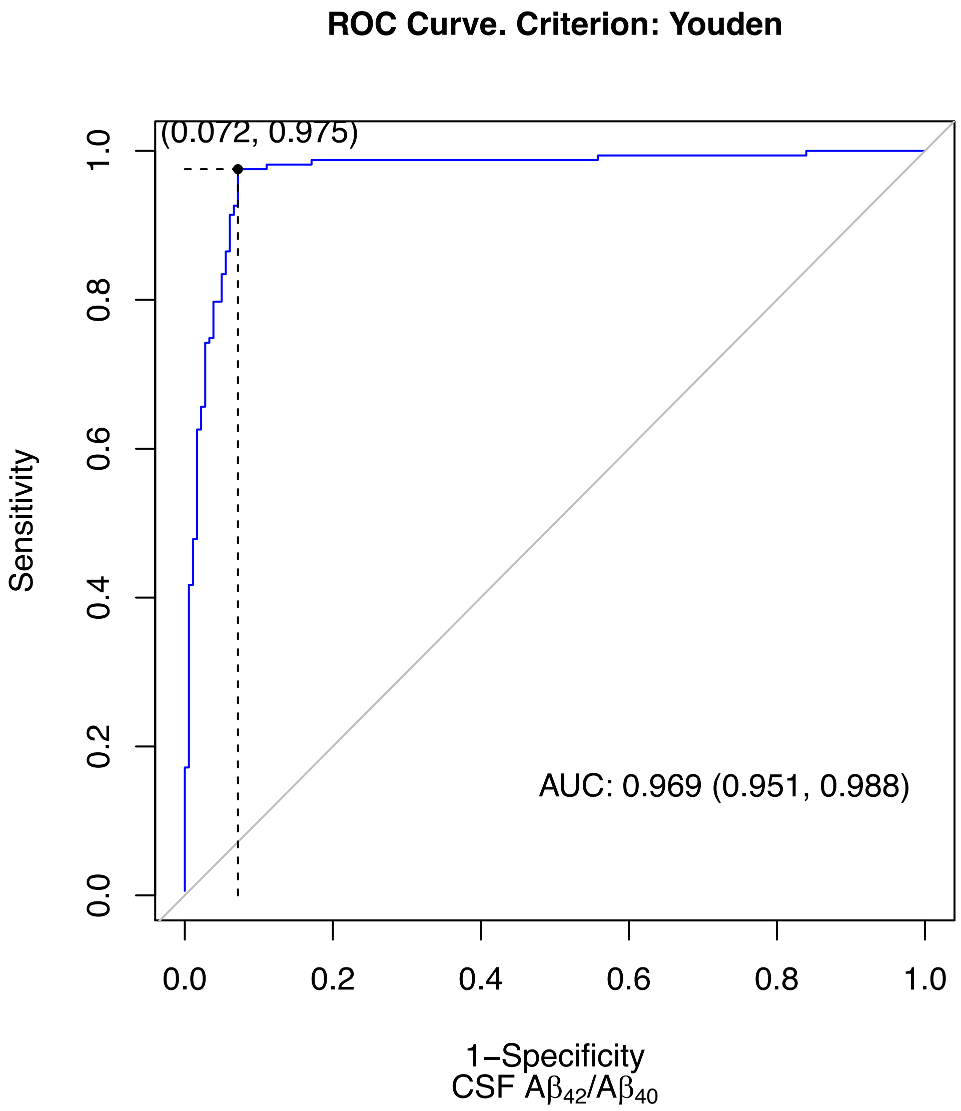

Supplementary figure 5. *The ROC analysis using the Youden index classifying 181 Aβ- ADNI CU participants and 163 Aβ+ ADNI MCI and AD patients as the endpoint to define the cutoff ⩽ 0.054 for CSF Aβ_{42}/Aβ_{40} ratio. AUC: 0.969 (95% CI, 0.951, 0.988).*

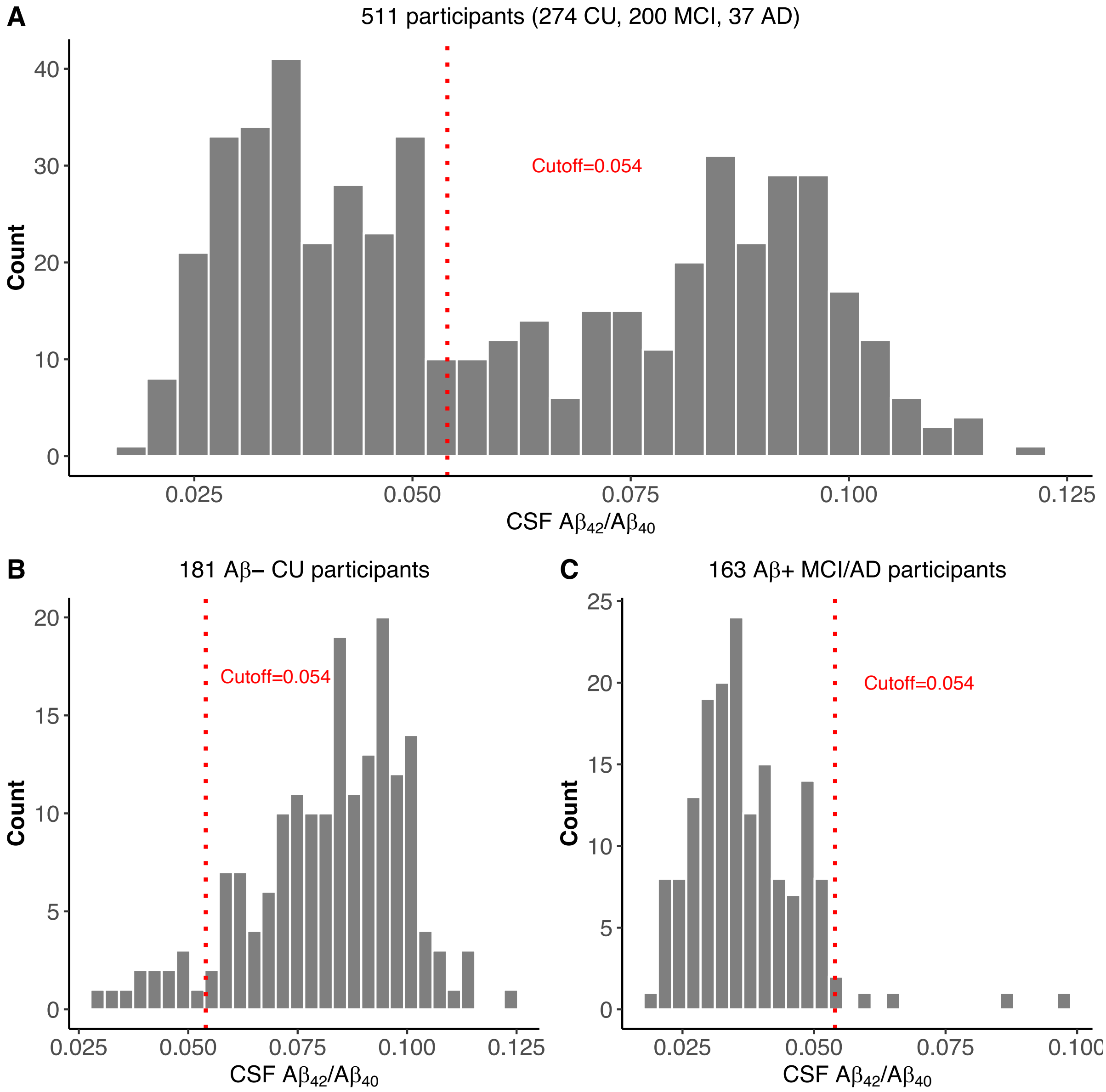

Supplementary figure 6. *Histograms of CSF $A\beta_{42}/A\beta_{40}$ for (A) all 511 ADNI participants, (B) 181 Aβ- ADNI CU participants and (C) 163 Aβ+ ADNI MCI and AD patients. Red dotted line is the 0.054 cutoff for the CSF $A\beta_{42}/A\beta_{40}$ ratio.*

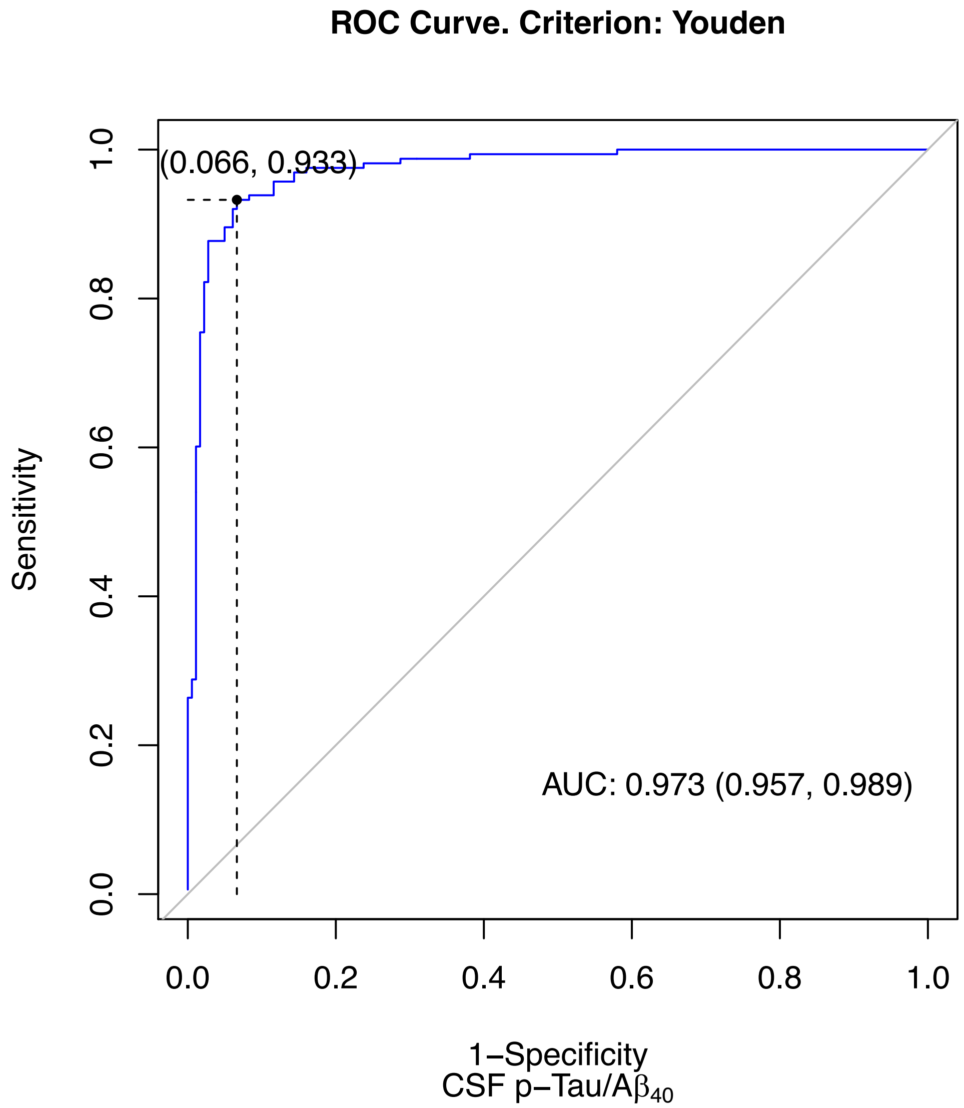

Supplementary figure 7. *The ROC analysis using the Youden index classifying 181 Aβ- ADNI CU participants and 163 Aβ+ ADNI MCI and AD patients as the endpoint to define the cutoff ⩾0.0012 for CSF p-Tau/Aβ$_{40}$ ratio. AUC: 0.973 (95% CI, 0.957, 0.989).*

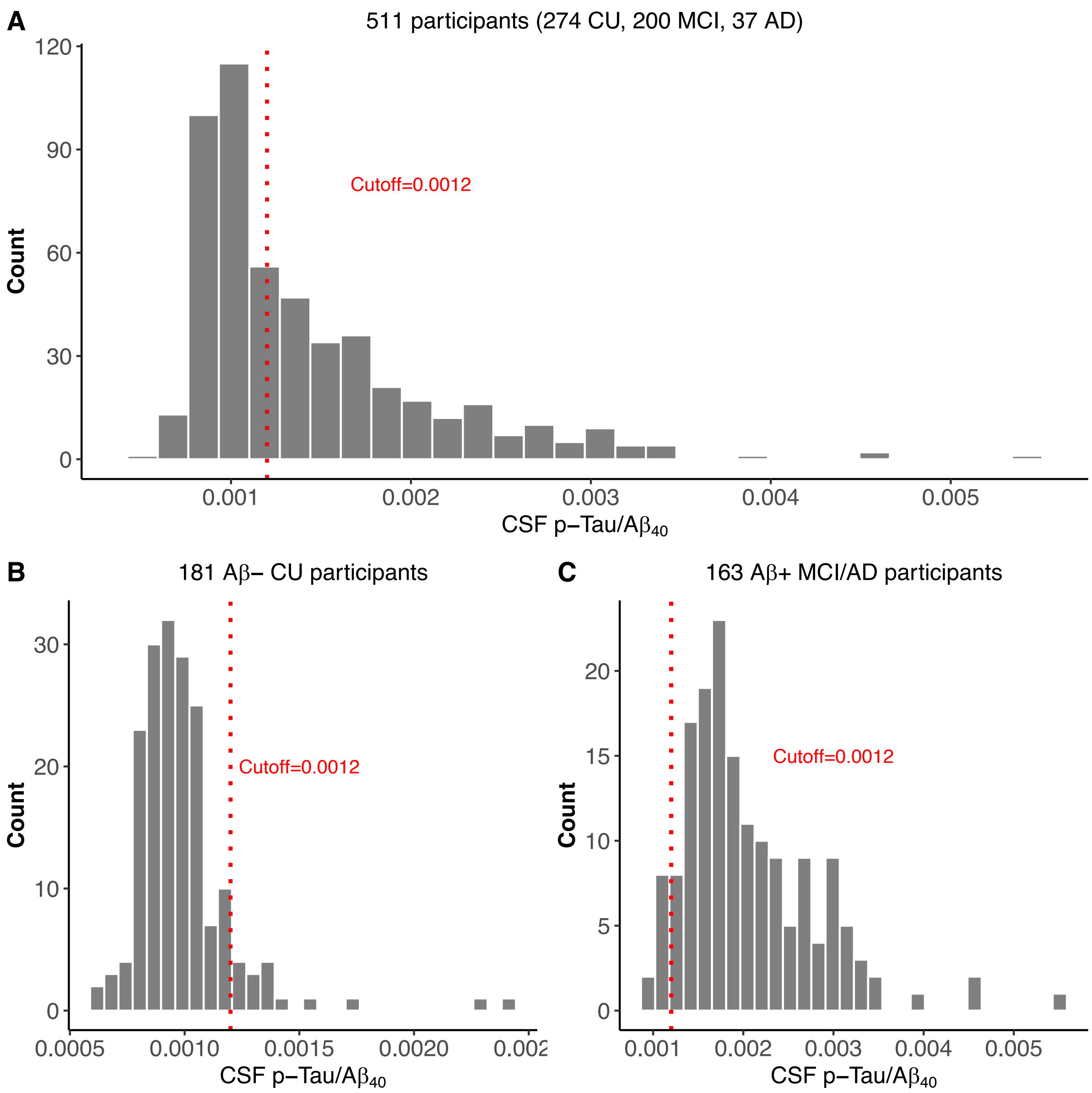

Supplementary figure 8. *Histograms of CSF p-Tau/Aβ_{40} for (A) all 511 ADNI participants, (B) 181 Aβ- ADNI CU participants and (C) 163 Aβ+ ADNI MCI and AD patients. Red dotted line is the 0.0012 cutoff for the CSF p-Tau/Aβ_{40} ratio.*

Analyses after excluding two individuals with high entorhinal tau

We noticed that one CSF+/PET- individual and one CSF+/PET+ individual had very high entorhinal FTP SUVRs (Fig.3A in Results), thus we repeated all the analyses after removing them from the dataset to ensure the main results were not caused by the several participants.

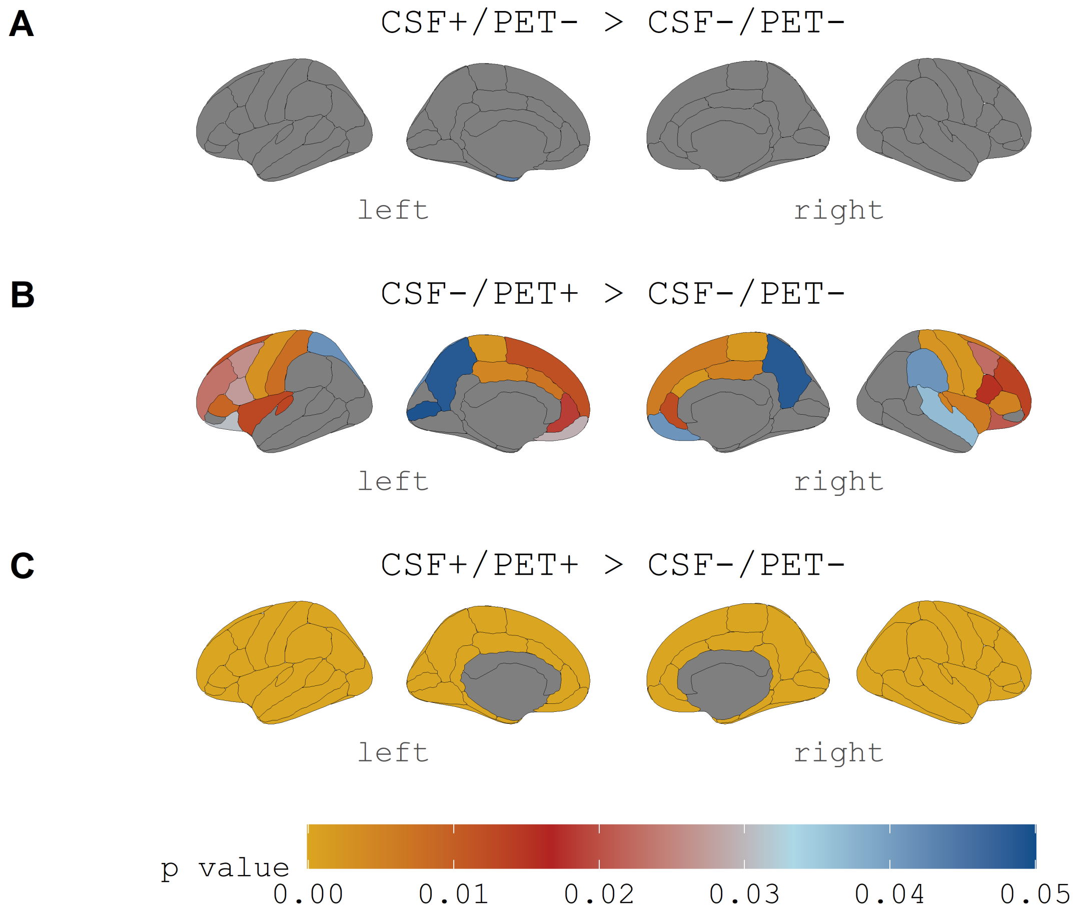

Supplementary figure 9. Significant cortical tau elevations of different CSF/PET groups categorized by CSF β-amyloid (Aβ) and Aβ PET after removing two individuals with high entorhinal tau. *Cortical regions with significant tau increases of (A) CSF+/PET-, (B)CSF-/PET+ and (C)CSF+/PET+ than the CSF-/PET- individuals, multiple comparisons correction was employed for 68 ROIs by using the Benjamini-Hochberg approach (FDR < 0.05) except for the comparison between CSF+/PET- group and Ref group.*

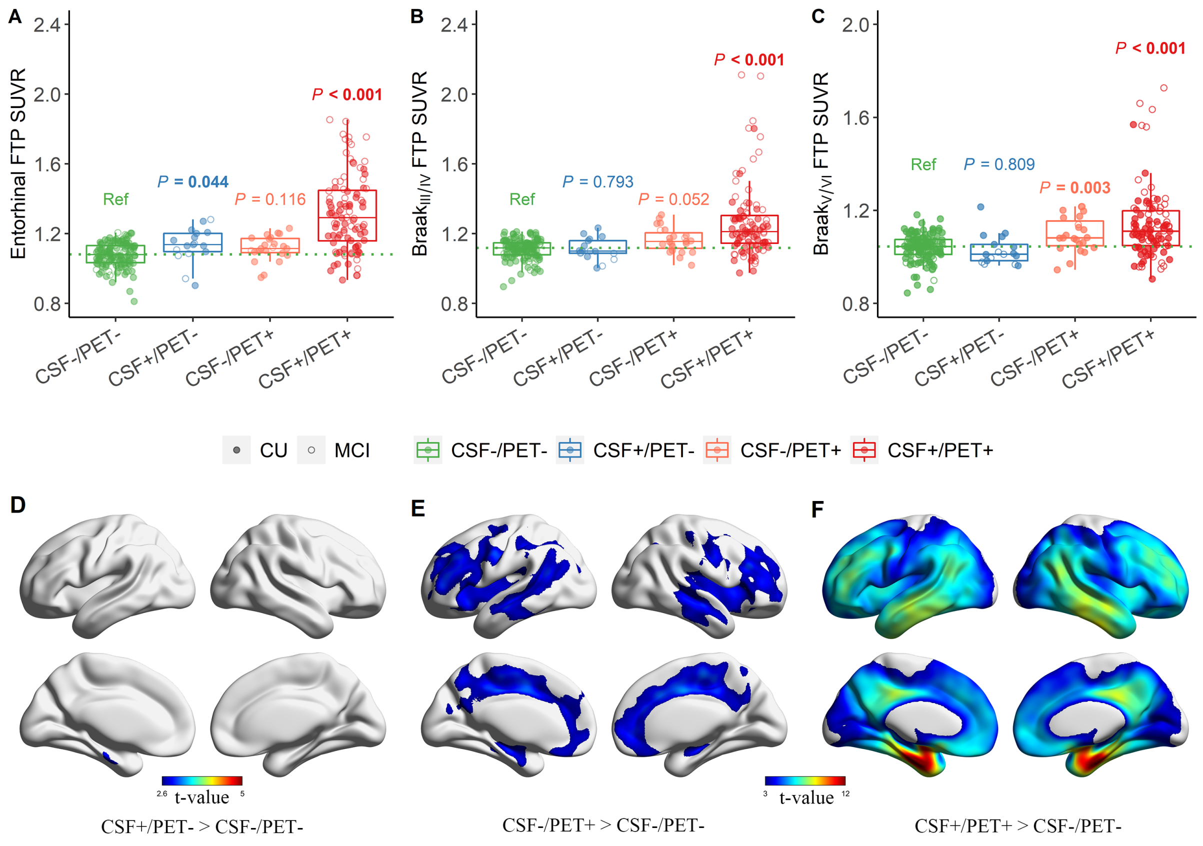

Supplementary figure 10. Comparison of cortical tau deposition between different CSF/PET groups after removing two individuals with high entorhinal tau. *Comparison of FTP SUVRs in (A) entorhinal, (B) Braak$_{III/IV}$, and (C) Braak$_{V/VI}$ among different CSF/PET groups. Voxel-wise comparisons of tau PET images of (D)CSF+/PET-, (E) CSF-/PET+, and (F) CSF+/PET+ with the Ref group. Two-sample t-tests, the comparison between CSF+/PET- group and the Ref group was presented by using a threshold p< 0.005 at the voxel level, while the other comparisons were presented by using a threshold p< 0.001 at the voxel level and with FWE corrected p < 0.05 at the cluster level.*

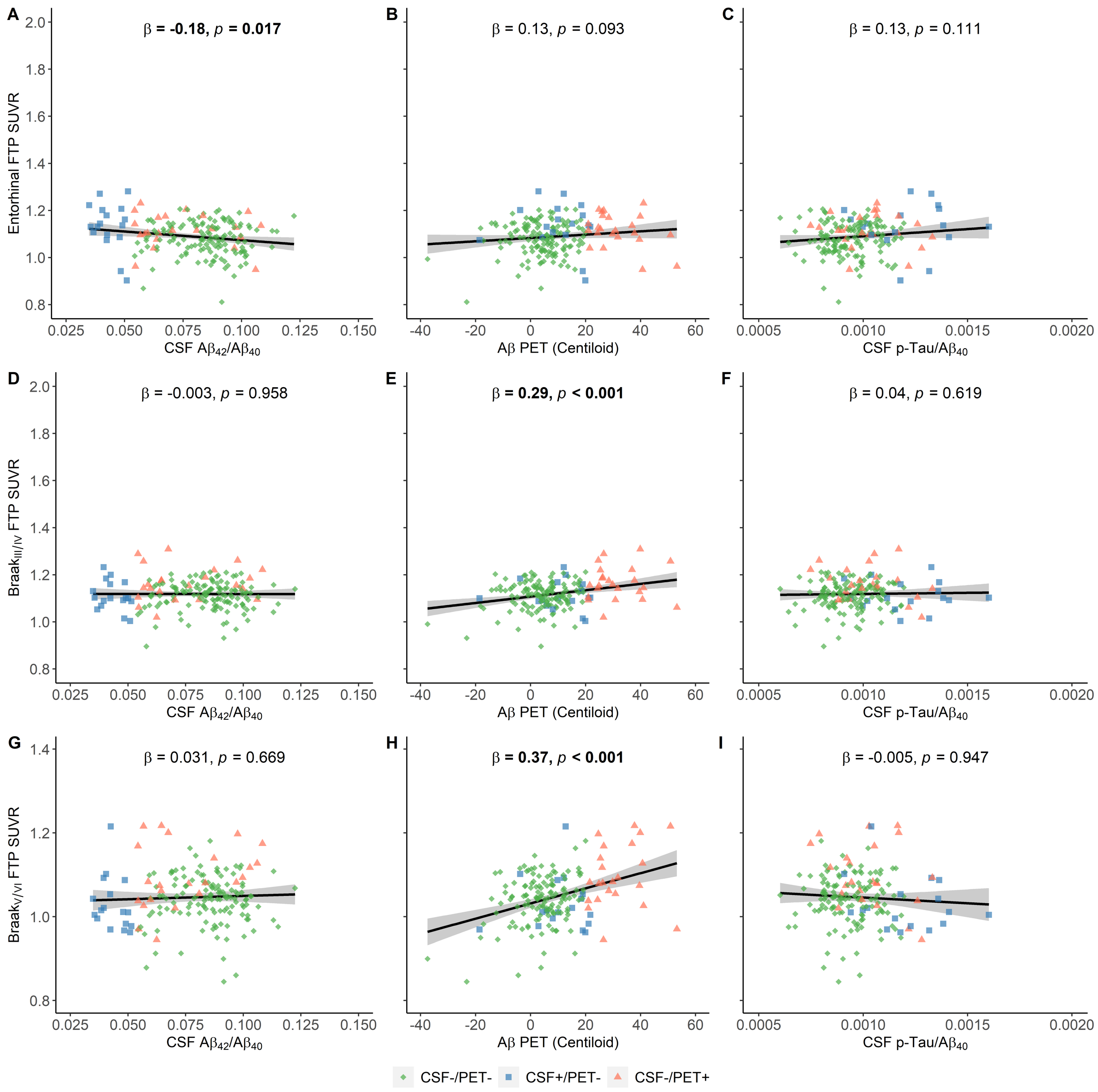

Supplementary figure 11. The association of cortical tau deposition with CSF $A\beta_{42}/A\beta_{40}$, Aβ PET and CSF p-Tau/$A\beta_{40}$ in early amyloidosis stage after removing two individuals with high entorhinal tau. *The association between entorhinal FTP SUVR and (A)CSF $A\beta_{42}/A\beta_{40}$, (B)Aβ-PET(Centiloid), and (C)CSF p-Tau/$A\beta_{40}$. The association between $Braak_{III/IV}$ FTP SUVR and (D)CSF $A\beta_{42}/A\beta_{40}$, (E) Aβ-PET(Centiloid), and (F)CSF p-Tau/$A\beta_{40}$. The association between $Braak_{V/VI}$ FTP SUVR and (G)CSF $A\beta_{42}/A\beta_{40}$, (H)Aβ-PET(Centiloid), and (I)CSF p-Tau/$A\beta_{40}$.*

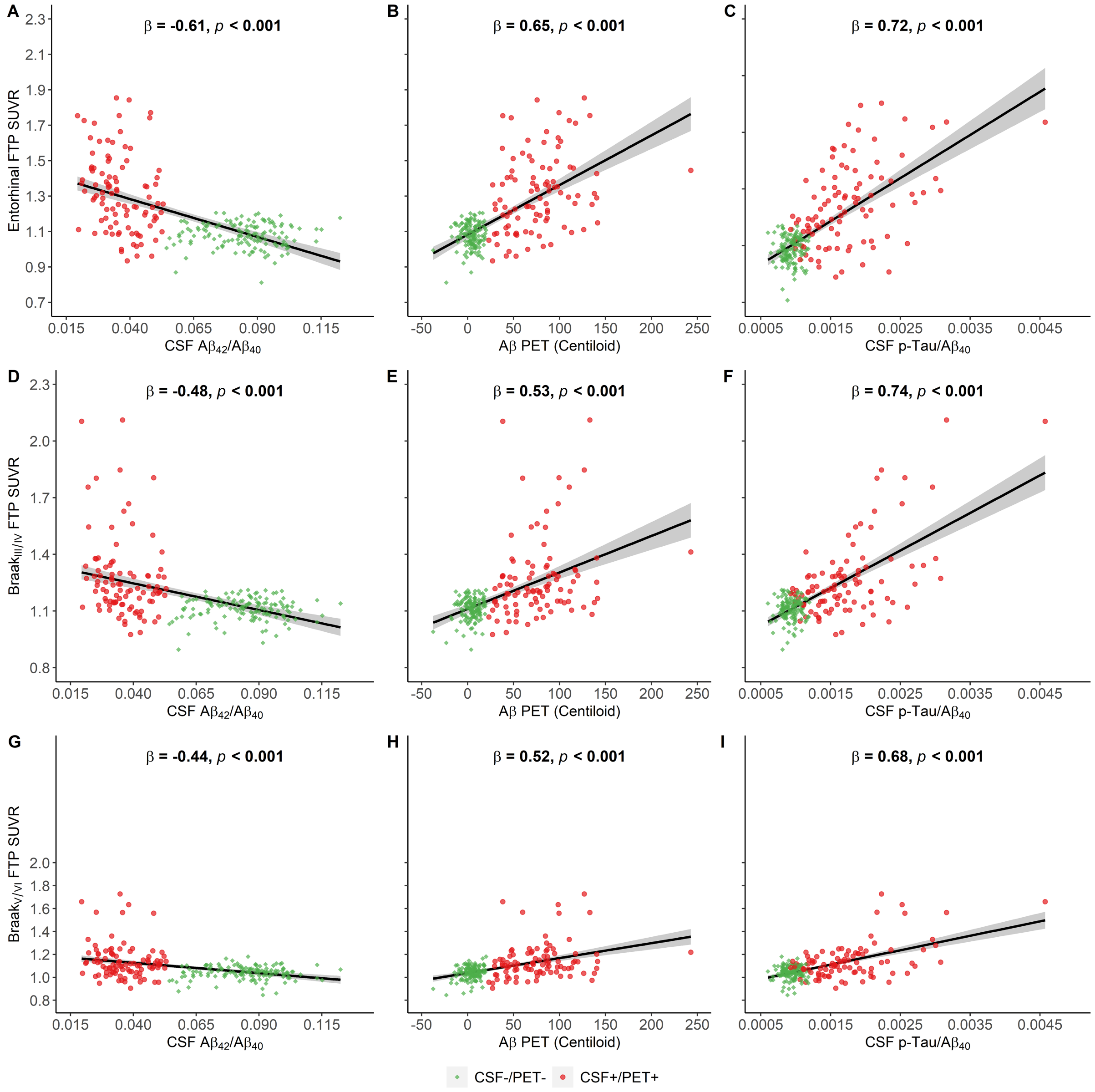

Supplementary figure 12. The association of cortical tau deposition with CSF $A\beta_{42}/A\beta_{40}$, Aβ PET and CSF p-Tau/$A\beta_{40}$ in late amyloidosis stage after removing two individuals with high entorhinal tau. *The association between entorhinal FTP SUVR and (A)CSF $A\beta_{42}/A\beta_{40}$, (B)Aβ-PET(Centiloid), and (C)CSF p-Tau/$A\beta_{40}$. The association between $Braak_{III/IV}$ FTP SUVR and (D)CSF $A\beta_{42}/A\beta_{40}$, (E)Aβ-PET(Centiloid), and (F)CSF p-Tau/$A\beta_{40}$. The association between Braak $_{V/VI}$ FTP SUVR and (G)CSF $A\beta_{42}/A\beta_{40}$, (H)Aβ-PET(Centiloid), and (I)CSF p-Tau/$A\beta_{40}$.*

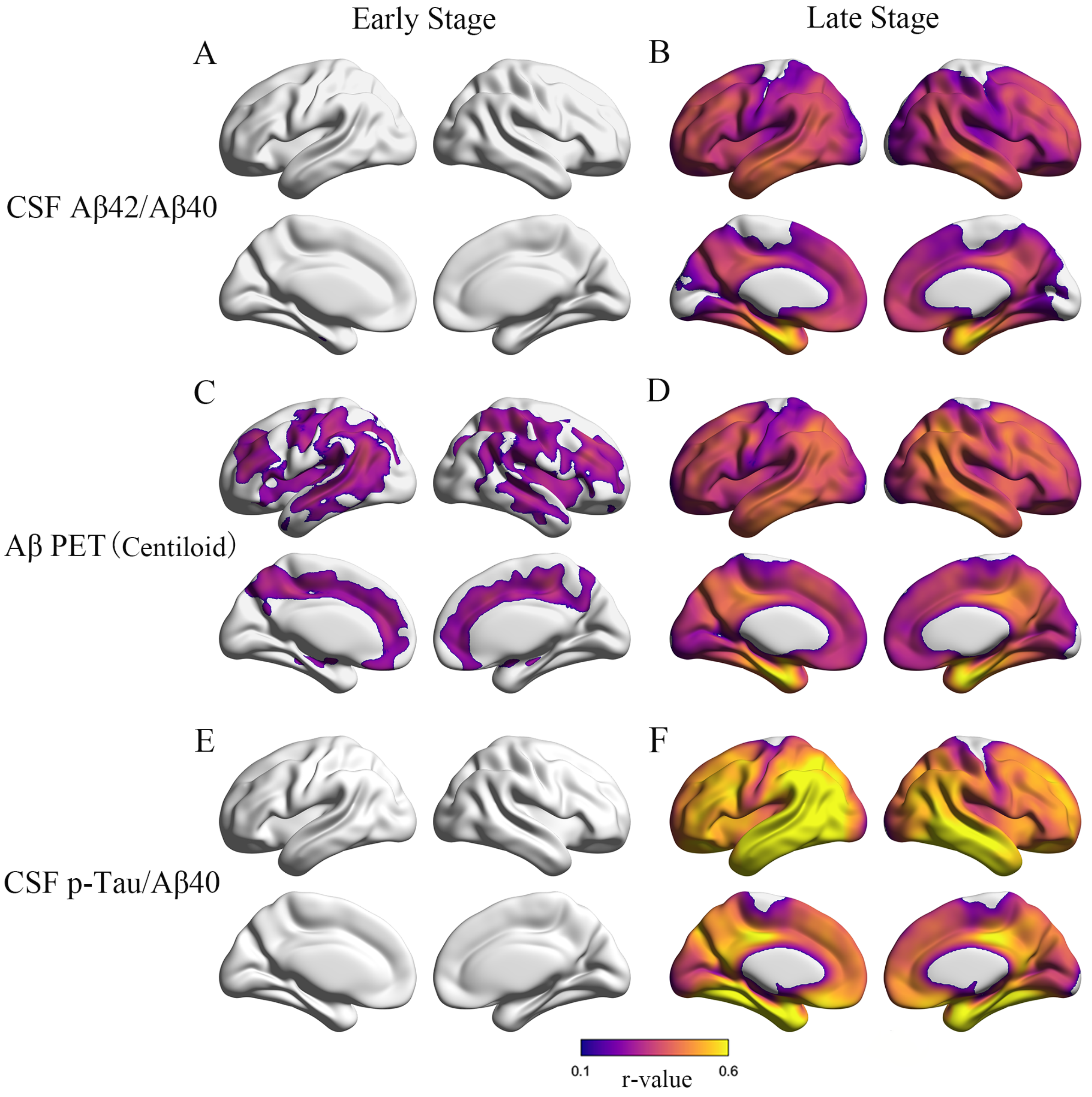

Supplementary figure 13. Voxel-wise analyses of cortical tau with CSF biomarkers and Aβ PET in early and late amyloidosis stages after removed two outliers. *Cortical regions with significant associations between FTP SUVR and* ***(A)*** *CSF* $A\beta_{42}/A\beta_{40}$*,* ***(C)*** *Aβ PET,* ***(E)*** *CSF p-Tau/*$A\beta_{40}$ *and in early amyloidosis stage. The voxel-wise correlation between CSF* $A\beta_{42}/A\beta_{40}$ *and FTP tau PET was presented as* $p < 0.005$ *at the voxel level without cluster correction. The other voxel-wise correlation results were presented with using a threshold* $p < 0.001$ *at the voxel level and with FWE corrected* $p < 0.05$ *at the cluster level. Cortical regions with significant associations between FTP SUVR and* ***(B)*** *CSF* $A\beta_{42}/A\beta_{40}$*,* ***(D)*** *Aβ PET,* ***(F)*** *CSF p-Tau/*$A\beta_{40}$ *and in the late amyloidosis stage. Results are shown using a threshold* $p < 0.001$ *at the voxel level and with FWE corrected* $p < 0.05$ *at the cluster level.*

Sensitivity analysis

Additionally, in order to exclude the influence of those individuals around the thresholds of CSF $A\beta_{42}/A\beta_{40}$ and Aβ PET SUVR, we also repeated all the analyses after excluding the borderline participants who were within ±5% of the CSF $A\beta_{42}/A\beta_{40}$ and Aβ PET SUVR (FBB and FBP) thresholds. We did the sensitivity analysis after excluding 30 CSF−/PET−, 10 CSF+/PET−, 16 CSF−/PET+ and 10 CSF+/PET+ borderline participants as showed in Supplementary Figure 14.

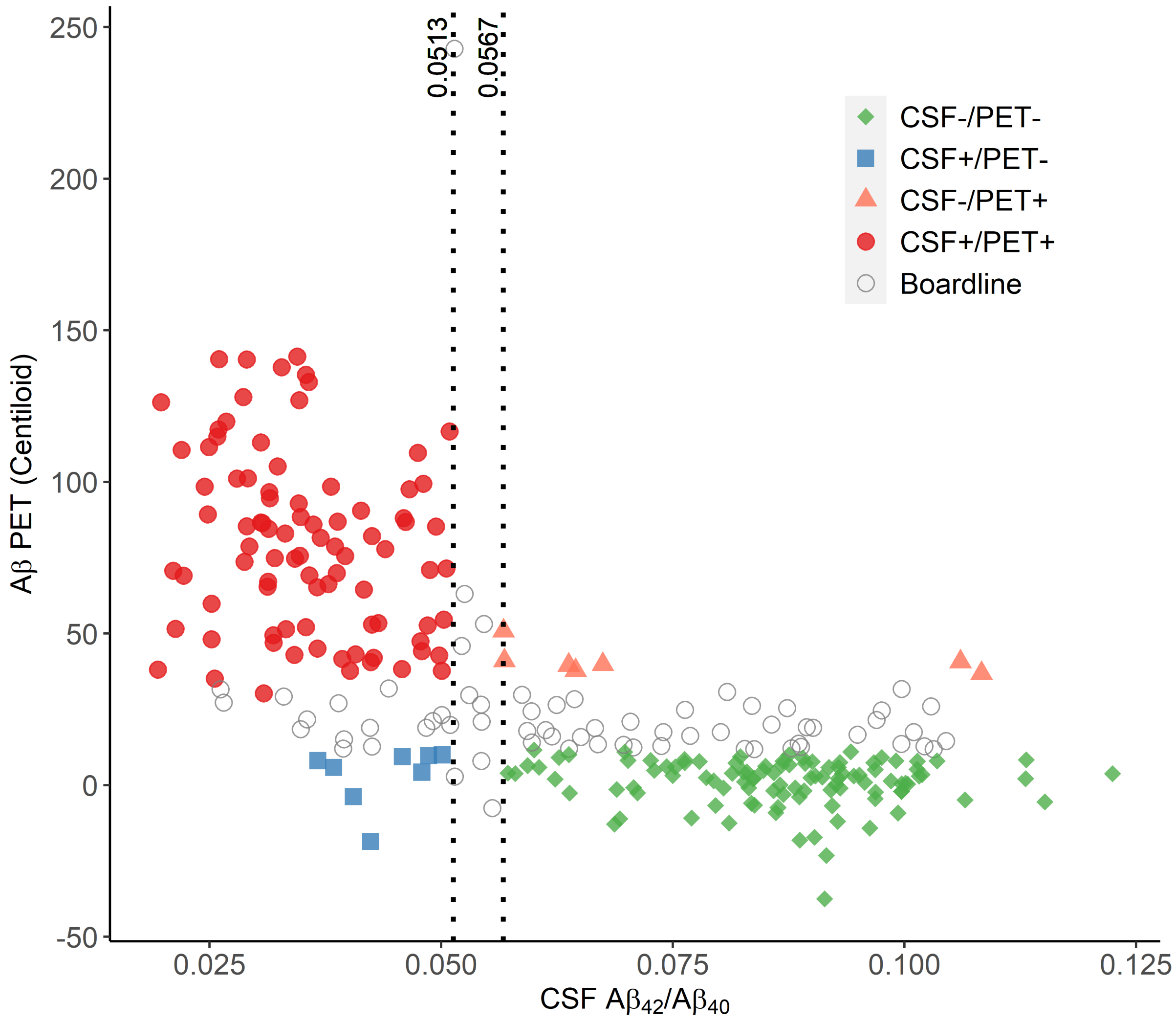

Supplementary figure 14. Illustration of different CSF/PET groups categorized by CSF β-amyloid (Aβ) and Aβ PET after excluding individuals around the borderline. *Notes: the borderline participants who were within ±5% of the Aβ PET were defined as those individuals whose SUVRs were within ±5% of SUVR cutoffs 1.11 (FBP) or 1.08 (FBB), but we showed them as Aβ PET Centiloids when we put FBP and FBB two different tracers together.*

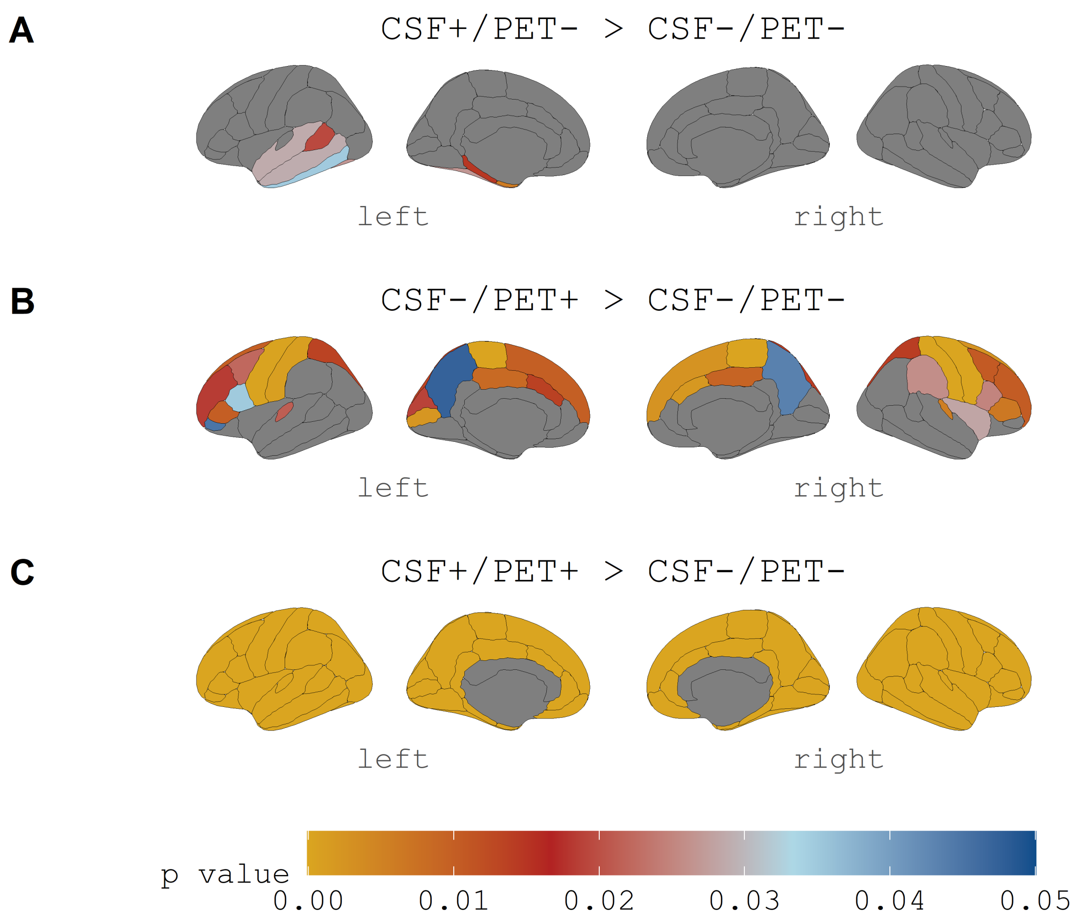

Supplementary figure 15. Significant cortical tau elevations of different CSF/PET groups categorized by CSF β-amyloid (Aβ) and Aβ PET after excluding individuals around the borderline. Cortical regions with significant tau increases of (A) CSF+/PET-, (B) CSF-/PET+ and (C)CSF+/PET+ groups than the CSF-/PET- group, multiple comparisons correction was employed for 68 ROIs by using the Benjamini-Hochberg approach (FDR < 0.05) except for the comparison between CSF+/PET- group and Ref group.

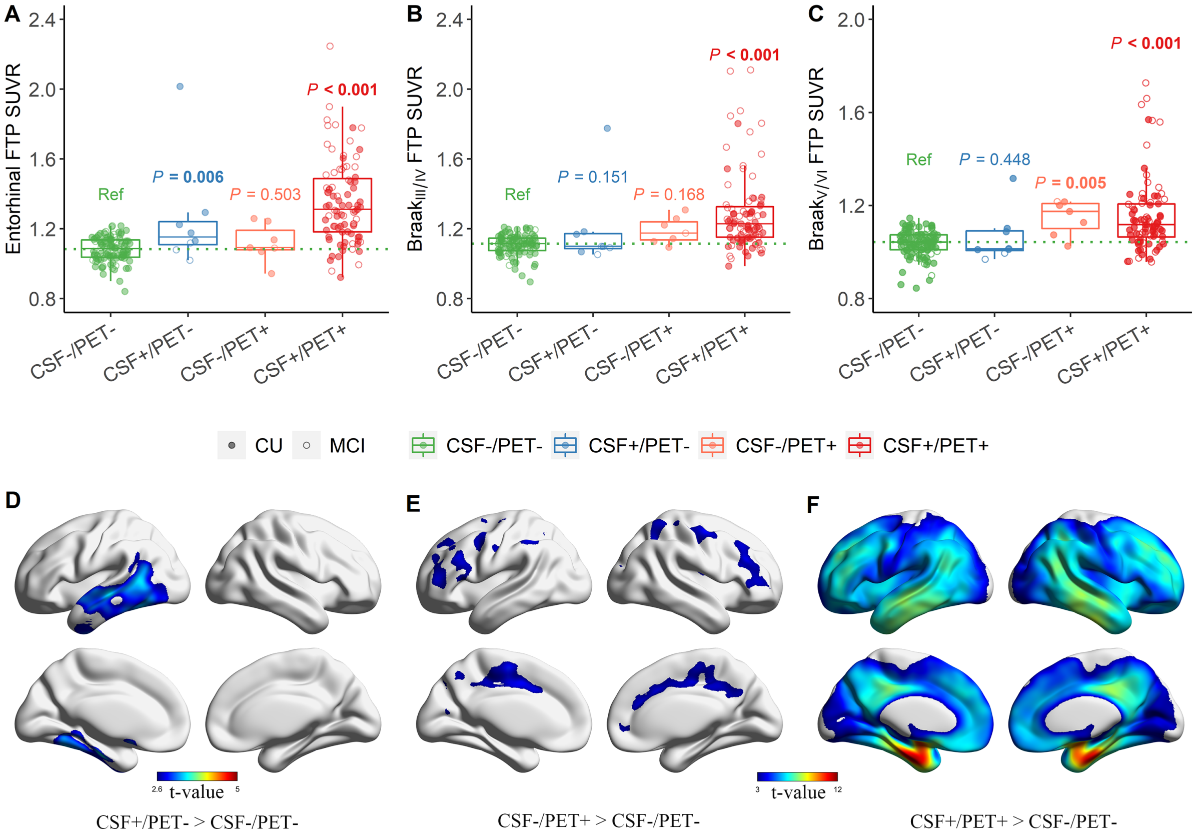

Supplementary figure 16. Comparison of cortical tau deposition between different CSF/PET groups after excluding individuals around the borderline. Comparison of FTP SUVRs in (A) entorhinal, (B) Braak$_{III/IV}$, and (C) Braak$_{V/VI}$. Voxel-wise comparisons of tau PET images of (D)CSF+/PET-, (E) CSF-/PET+, and (F) CSF+/PET+ with the CSF-/PET- (Ref) group. Two-sample t-tests, the comparison between CSF+/PET- group and the Ref group was presented by using a threshold p < 0.005 at the voxel level, while the other comparisons were presented by using a threshold p < 0.001 at the voxel level and with FWE corrected p < 0.05 at the cluster level.

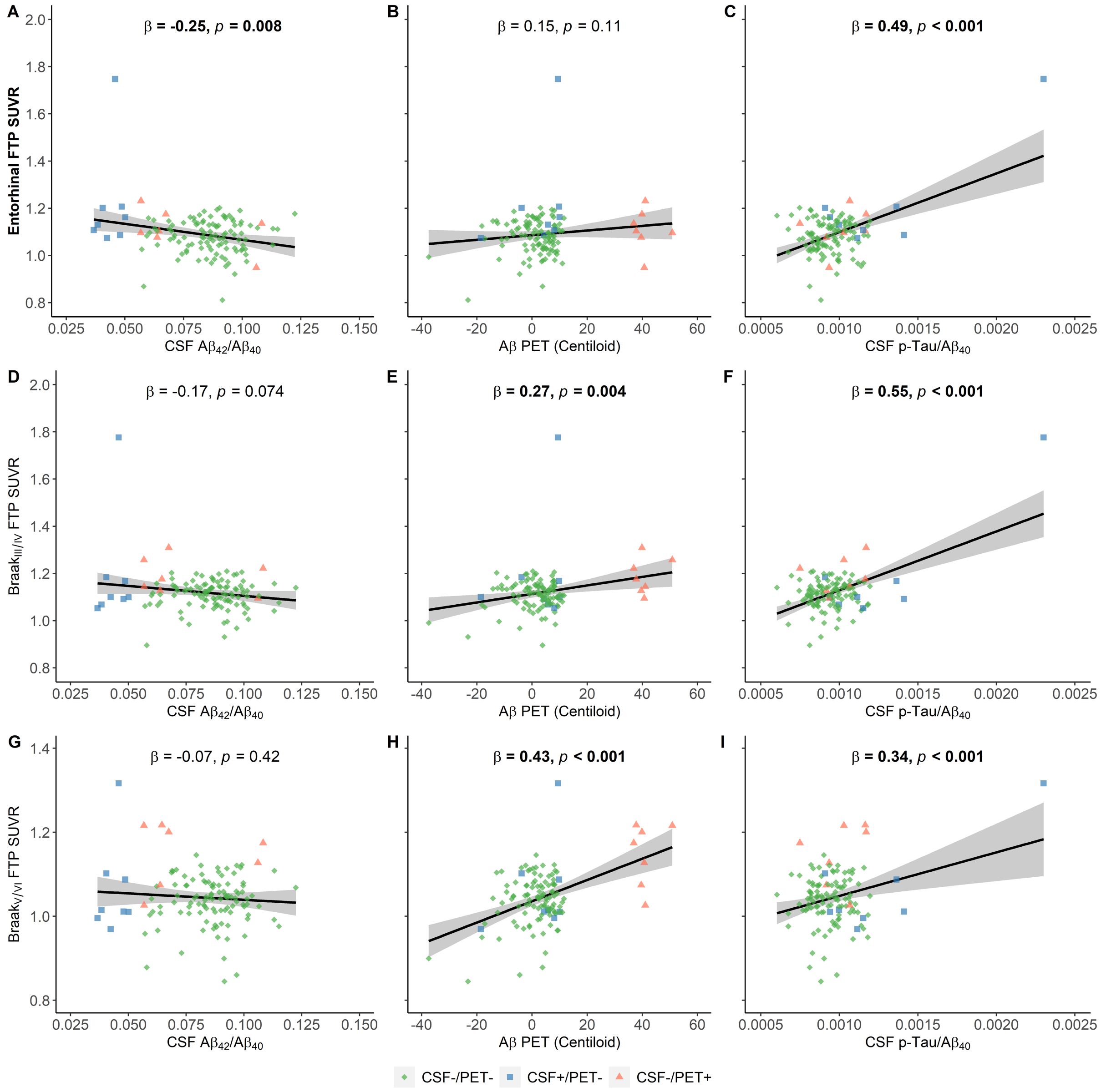

Supplementary figure 17. The association of cortical tau deposition with CSF $A\beta_{42}/A\beta_{40}$, Aβ PET and CSF p-Tau/$A\beta_{40}$ in early amyloidosis stage after excluding individuals around the borderline. The association of entorhinal FTP SUVR with (A)CSF $A\beta_{42}/A\beta_{40}$, (B)Aβ-PET(Centiloid), and (C)CSF p-Tau/$A\beta_{40}$. The association of $\text{Braak}_{\text{III/IV}}$ FTP SUVR with (D)CSF $A\beta_{42}/A\beta_{40}$, (E)Aβ-PET(Centiloid), and (F)CSF p-Tau/$A\beta_{40}$. The association of $\text{Braak}_{\text{V/VI}}$ FTP SUVR with (G)CSF $A\beta_{42}/A\beta_{40}$, (H)Aβ-PET(Centiloid), and (I)CSF p-Tau/$A\beta_{40}$.

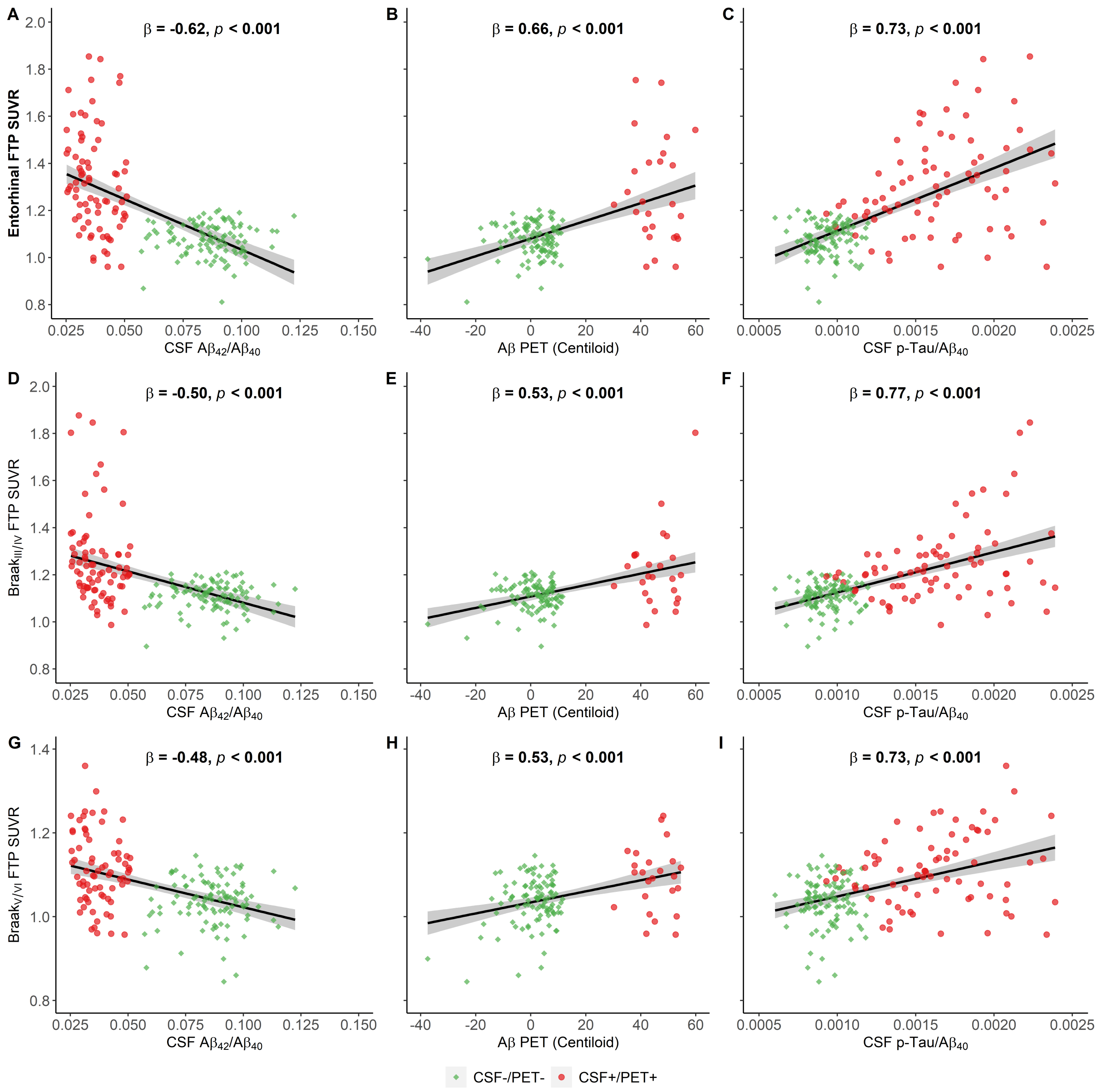

Supplementary figure 18. The association of cortical tau deposition with CSF $A\beta_{42}/A\beta_{40}$, Aβ PET and CSF p-Tau/$A\beta_{40}$ in late amyloidosis stage after excluding individuals around the borderline. The association between entorhinal FTP SUVR and (A)CSF $A\beta_{42}/A\beta_{40}$, (B)Aβ-PET(Centiloid), and (C)CSF p-Tau/$A\beta_{40}$. The association between $Braak_{III/IV}$ FTP SUVR and (D)CSF $A\beta_{42}/A\beta_{40}$, (E)Aβ-PET(Centiloid), and (F)CSF p-Tau/$A\beta_{40}$. The association between $Braak_{V/VI}$ FTP SUVR and (G)CSF $A\beta_{42}/A\beta_{40}$, (H)Aβ-PET(Centiloid), and (I)CSF p-Tau/$A\beta_{40}$.

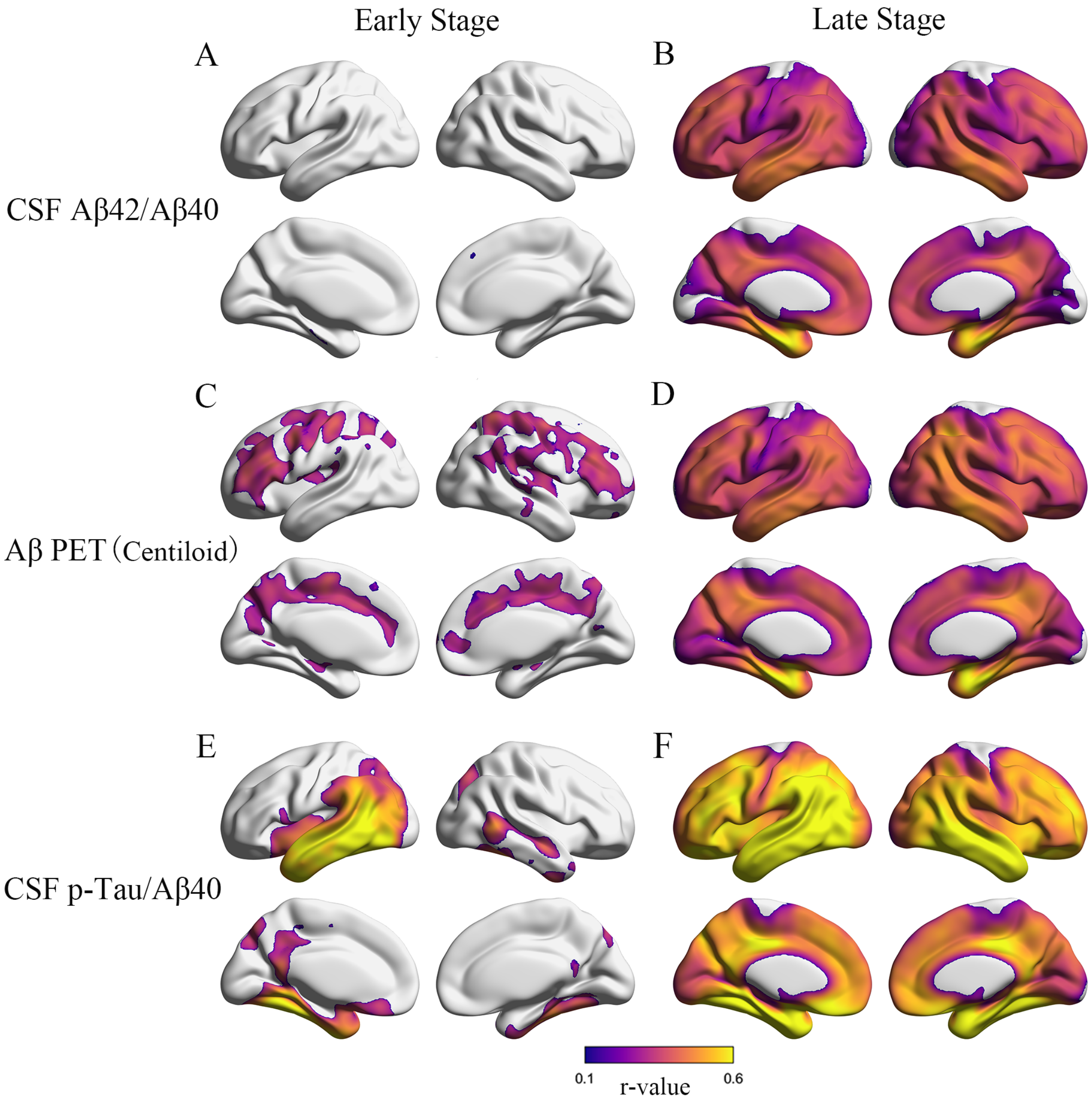

Supplementary figure 19. Voxelwise analyses of cortical tau with CSF biomarkers and Aβ PET in early and late amyloidosis stages after excluding individuals around the borderline. Cortical regions with significant associations between FTP SUVR and **(A)** CSF $A\beta_{42}/A\beta_{40}$, **(C)** Aβ PET, **(E)** CSF p-Tau/$A\beta_{40}$ and in early amyloidosis stage. The voxel-wise correlation between CSF $A\beta_{42}/A\beta_{40}$ and FTP tau PET was presented as $p < 0.005$ at the voxel level without cluster correction. The other voxel-wise correlation results were presented with using a threshold $p < 0.001$ at the voxel level and with FWE corrected $p < 0.05$ at the cluster level. Cortical regions with significant associations between FTP SUVR and **(B)** CSF $A\beta_{42}/A\beta_{40}$, **(D)** Aβ PET, **(F)** CSF p-Tau/$A\beta_{40}$ and in the late amyloidosis stage. Results are shown using a threshold $p < 0.001$ at the voxel level and with FWE corrected $p < 0.05$ at the cluster level.
